# A haplotype-led approach to increase the precision of wheat breeding

**DOI:** 10.1038/s42003-020-01413-2

**Published:** 2020-11-25

**Authors:** Jemima Brinton, Ricardo H. Ramirez-Gonzalez, James Simmonds, Luzie Wingen, Simon Orford, Simon Griffiths, 10 Wheat Genome Project, Georg Haberer, Manuel Spannagl, Sean Walkowiak, Curtis Pozniak, Cristobal Uauy

**Affiliations:** 1 John Innes Centre, Norwich Research Park, Norwich, NR4 7UH UK; 2 Helmholtz Zentrum München – Research Center for Environmental Health, Neuherberg, Germany; 3 University of Saskatchewan, Crop Development Centre, Saskatoon, Saskatchewan Canada; 4 Grain Research Laboratory, Canadian Grain Commission, Winnipeg, MB Canada; 5 Present Address: Department of Natural Capital and Plant Health, Royal Botanic Gardens, Kew, Richmond, UK

**Keywords:** Plant breeding, Plant genetics

## Abstract

Crop productivity must increase at unprecedented rates to meet the needs of the growing worldwide population. Exploiting natural variation for the genetic improvement of crops plays a central role in increasing productivity. Although current genomic technologies can be used for high-throughput identification of genetic variation, methods for efficiently exploiting this genetic potential in a targeted, systematic manner are lacking. Here, we developed a haplotype-based approach to identify genetic diversity for crop improvement using genome assemblies from 15 bread wheat (*Triticum aestivum*) cultivars. We used stringent criteria to identify identical-by-state haplotypes and distinguish these from near-identical sequences (~99.95% identity). We showed that each cultivar shares ~59 % of its genome with other sequenced cultivars and we detected the presence of extended haplotype blocks containing hundreds to thousands of genes across all wheat chromosomes. We found that genic sequence alone was insufficient to fully differentiate between haplotypes, as were commonly used array-based genotyping chips due to their gene centric design. We successfully used this approach for focused discovery of novel haplotypes from a landrace collection and documented their potential for trait improvement in modern bread wheat. This study provides a framework for defining and exploiting haplotypes to increase the efficiency and precision of wheat breeding towards optimising the agronomic performance of this crucial crop.

## Abstract (web summary)

Brinton, Uauy and colleagues utilize genomic data from the 10+ Wheat Genome Project to develop a useful tool for studying and generating new wheat cultivars. This framework uses advanced exploitation of wheat haplotypes to bring newfound precision and efficiency to wheat breeding.

## Introduction

Crop breeding involves assembling desired combinations of traits to generate improved cultivars^1^. Most of these traits are governed by genomic regions defined by underlying genetic variation (e.g. SNPs, indels, copy number variations). These genomic regions are often co-inherited as blocks of variation, or haplotypes, which are the effective units of selection by breeders. Most breeders currently use one or a few SNP markers to tag these haplotypes and introduce novel traits from diverse germplasm into modern cultivars^2,3^. Although valuable, these markers are often not causal but are simply associated with the trait of interest, providing limited information about the surrounding sequences. This can result in the selection of false-positive and false-negative individuals, which limits the efficient use of genetic diversity within breeding programmes^4^. Genomics provides a powerful means to define these haplotypes more precisely and implement targeted approaches to identify and exploit novel genetic variation efficiently to enhance crop performance. This is especially relevant given that the genetic diversity of many crops, including self-pollinating hexaploid bread wheat, has been severely constrained during the course of domestication and subsequent pure-line breeding^5,6^.

Here, we defined haplotype blocks in hexaploid bread wheat using genome assemblies of cultivars representing modern-day diversity across wheat breeding programmes^7^. We used chromosome-scale genome assemblies corresponding to 9 wheat lines (ArinaLrFor, Jagger, Julius, Lancer, Landmark, Mace, Norin61, Stanley, SY-Mattis)^7^ and the Chinese Spring RefSev1.0 assembly^8^, alongside 5 scaffold-level assemblies corresponding to cultivars Cadenza, Claire, Paragon, Robigus and Weebill^7^. Thirteen of these 15 lines are considered cultivars (cultivated varieties of wheat), whereas ArinaLrFor is a line derived from cultivar Arina and Chinese Spring is a landrace collected in the early 1900s from China. To avoid multiple designations, for the purposes of this study we will refer to all 15 lines as cultivars.

## Results and discussion

### Defining haplotype blocks

**Fig. 1:**
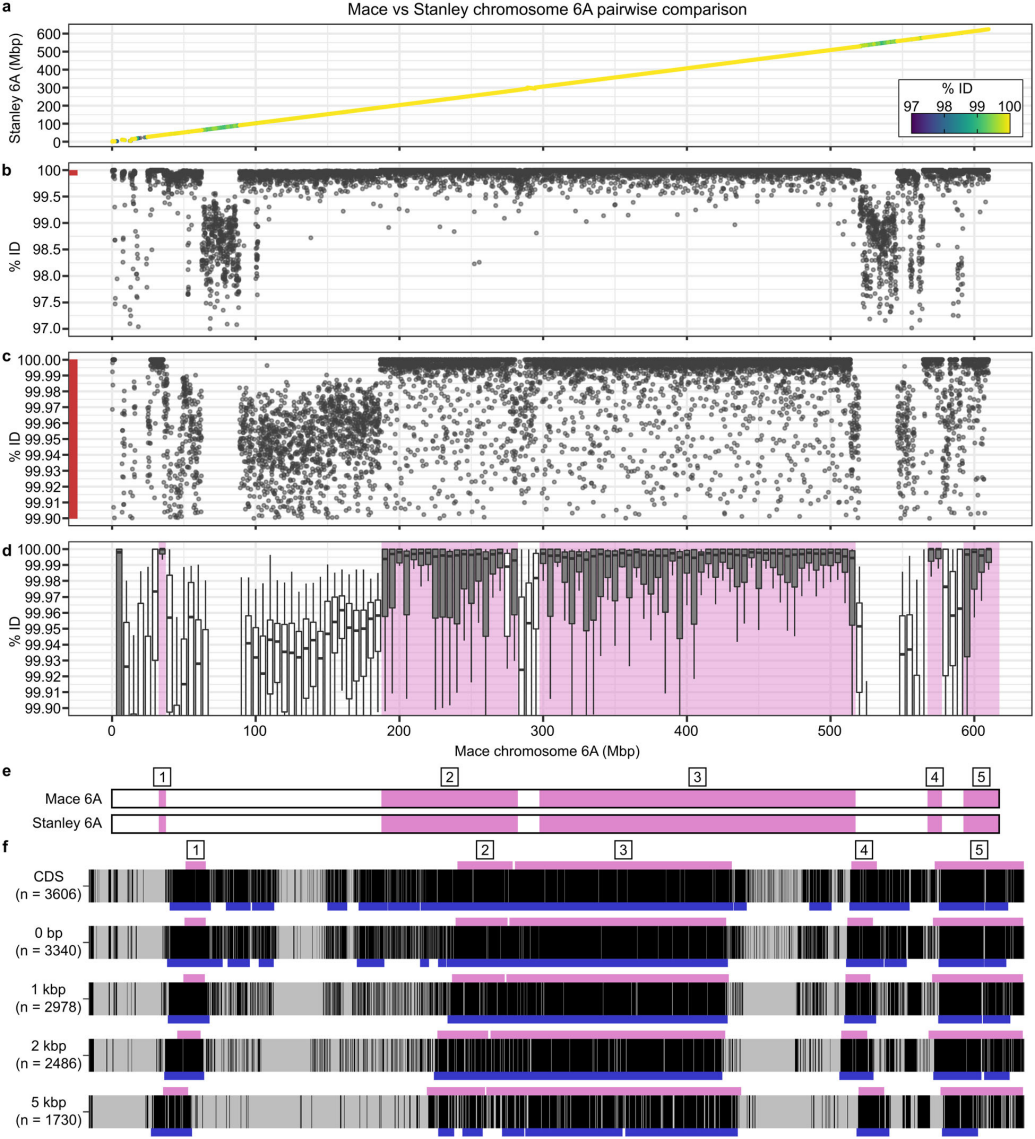
Definition of haplotype blocks using Mace and Stanley chromosome 6A as an illustrative example. **a** NUCmer alignments (≥20 Kbp length) between chromosome 6A (chr6A) of the Mace and Stanley assemblies. Each data point represents a single pairwise alignment, with the position corresponding to the midpoint of the alignment with respect to the Mace (*x*-axis) and Stanley (*y*-axis) assemblies. **b** % sequence identity of the pairwise NUCmer alignments between chr6A of Mace and Stanley in (**a**) with respect to the position in the Mace assembly. Each data point represents a single pairwise alignment. **c** Close-up view of (**b**) (red bar) between 99.90 and 100.00 % sequence identity. **d** Boxplots show the pairwise NUCmer alignments between chr6A of Mace and Stanley from (**b**) grouped into 5-Mbp bins. Grey-filled boxplots indicate bin median ≥99.99% sequence identity. Pink-shaded regions indicate intervals defined as haplotype blocks using the NUCmer-based approach (adjacent bins with median ≥99.99% stitched together allowing for two bins below the threshold, see “Methods”). **e** Diagrammatic representation of haplotype blocks between Mace and Stanley on chr6A called in (**d**). Mace physical position is a common axis for (**a**–**e**). **f** Pairwise BLAST alignments of genes on chromosome 6A in Mace and Stanley based on projections of RefSeqv1.1 high-confidence gene models (ordered by RefSeqv1.1 coordinates). The amount of flanking sequence included is indicated on the left, *n* indicates number of genes (sequences with Ns were excluded). Black = 100% identity, grey = <100% identity. Numbered boxes/pink rectangles above the heatmaps show the locations of NUCmer-based haplotype blocks defined in (**e**). Blue boxes below the heatmaps are haplotype blocks called based on BLAST alignments (25-gene sliding window; see “Methods”).

To define haplotypes, we first generated whole-chromosome pairwise alignments between all 15 cultivars using NUCmer, excluding pairwise comparisons between scaffold-level assemblies (Fig. 1a, b; chromosome 6A of Mace and Stanley shown as an illustrative example). We filtered the NUCmer output to retain only alignments ≥20 Kbp in length, thereby excluding shorter alignments between non-syntenic retrotransposons which have a median size of 9584 bp in wheat^9^. For each alignment, we calculated the percentage sequence identity ((number of mismatches/length of alignment)*100) and plotted these values across the chromosome (Fig. 1b). We identified physical regions that were identical-by-state between pairs of cultivars, with most pairwise alignments having sequence identities of 100%, whereas other physical intervals were ‘near-identical’, with most pairwise alignments having >99.90% sequence identity but not reaching 100% (i.e. more than 1 mismatch per 10 Kbp; Fig. 1b,c, Supplementary Fig. 1a). The presence of near-identical sequences (<99.99% sequence identity) is consistent with the expected sequence divergence in wheat haplotypes over the past 10,000 years (99.968%, see “Methods”), whereas the physical intervals with <99.5% sequence identity were consistent with introgressions from more distant wild wheat relatives^7,10,11^. For each set of pairwise alignments between cultivars, we calculated the median sequence identity of alignments in 5-Mbp bins across chromosomes. We defined identical-by-state haplotype blocks between pairs of cultivars as physical regions (bins) with ≥99.99% median sequence identity, stitching together adjacent bins exceeding the ≥99.99% threshold (Fig. 1d). This 99.99% cut-off accommodated Ns within alignments and sequencing errors due to low-complexity sequences (Supplementary Fig. 1b).

Existing methods to define haplotypes typically use genotyping data (e.g. from SNP arrays or re-sequencing) and are based on linkage disequilibrium or a fixed window size of adjacent markers^12,13^. A maximum level of diversity is allowed (e.g. 1–3%) to account for genotyping errors, while still grouping highly similar lines into the same haplotype group. Our results provide evidence that this level of divergence is not sufficiently stringent to accurately differentiate haplotypes in wheat and possibly other crops. However, the differentiation of haplotypes (median ≥ 99.99%) and ‘near-identical’ sequences (median < 99.99%) may not be relevant for breeding. To investigate this potential issue, we examined the 300 Kbp interval surrounding the two semi-dwarfing *Reduced Height* genes (*RHT-B1* and *RHT-D1*), which were the genetic basis for the Green Revolution in wheat^14^. We identified five *RHT-B1* and four *RHT-D1* haplotypes in the 15 sequenced cultivars. The Green Revolution *RHT* mutant haplotypes had ~99.96% sequence identity with all wild-type haplotypes, with alignments extending across >99% of the 300 Kbp region (Supplementary Fig. 2a). This result suggests that the ability to distinguish between identical-by-state and near-identical sequences will be essential for accurately classifying haplotypes for crop improvement.

As the NUCmer-based approach does not allow for direct comparisons between the five scaffold-level assemblies, we used a complementary method based on a sliding window approach using pairwise BLAST alignments of reference gene model projections to all assemblies^7^ (see “Methods” for details). To select parameters for defining haplotype blocks using gene-based BLAST alignments we performed a precision-recall analysis using the NUCmer-defined haplotypes as ground truth, testing a range of sliding window sizes (10, 15, 20, 25 and 30 genes) and flanking sequence surrounding genes (CDS only and gDNA ± 0, 1-, 2- or 5-Kbp) (Supplementary Fig. 3a). Importantly, we observed that using only the coding sequence (CDS) or exon–intron sequences of genes to define haplotypes was not sufficient to distinguish between haplotype blocks and near-identical sequences, whilst including additional flanking sequence improved the resolution (i.e. higher precision). However, loss of data due to Ns increased substantially with increasing flanking sequence, reducing the power to identify haplotype blocks (i.e. lower recall). We therefore selected 2-Kbp flanking sequence, which provided a balance between accuracy and loss of data (Fig. 1e, f, Supplementary Fig. 3a). Similarly, the precision increased with increasing sliding window size whilst the recall decreased. We selected a 25 gene sliding window as this provided a balance between precision and recall (Supplementary Fig. 3a). To generate the final set of genome-wide haplotype blocks, we combined the blocks called from the NUCmer and BLAST approaches). In total, we identified 4485 pairwise haplotype blocks genome wide using 5-Mbp bins (Supplementary Fig. 3b, Supplementary Data 2). We also performed this analysis using 2.5- and 1-Mbp bins to provide finer resolution (7578 and 17,693 pairwise haplotype blocks, respectively; Supplementary Fig. 3b, Supplementary Data 2).

### Genome-wide haplotype characterisation

**Fig. 2:**
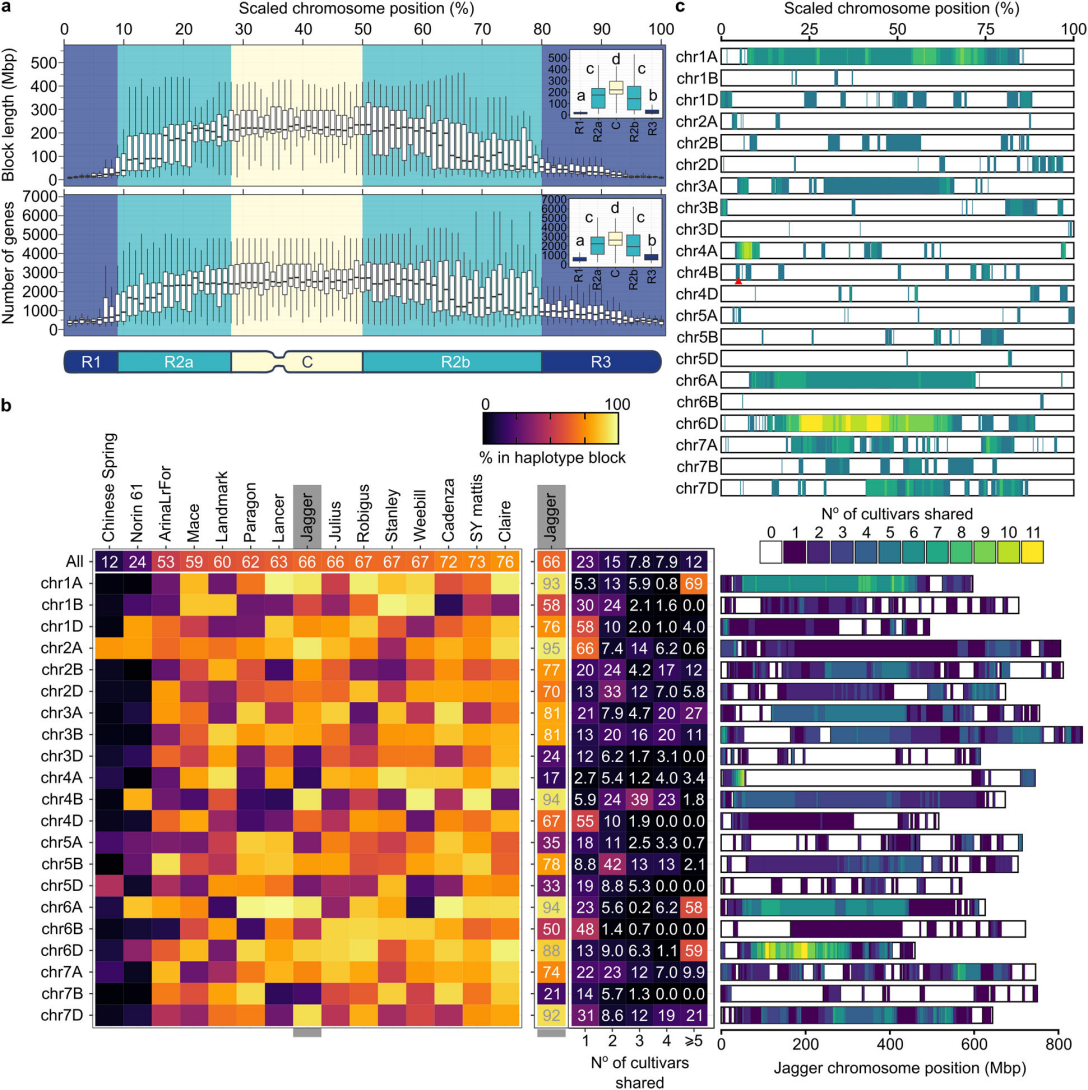
Genome-wide characterisation of haplotype blocks. **a** Length (upper) and gene number (middle) of haplotype blocks sampled at 500 Kbp intervals across all 21 chromosomes (positions scaled to % of maximum chromosome length). Boxplots show distributions of 1% bins. Insets show boxplots for chromosomal compartments (bottom) based on recombination (R1 + R3 > R2a + R2b > C)^15^. Pairwise Wilcox test with Benjamini–Hochberg multiple testing correction was used for statistical analysis. **b** Leftmost heatmap shows % of the genome (top row) or chromosomes contained within haplotype blocks for all cultivars. Jagger values are highlighted (middle), with a breakdown of how many other cultivars share these Jagger haplotype blocks. Rightmost panel shows the physical positions of Jagger haplotype blocks. **c** Summary of highly conserved haplotype blocks across all cultivars (shared with ≥ 5 cultivars; positions scaled to % of maximum chromosome length per cultivar). Values based on haplotype blocks called using 5-Mbp bins. *RHT-B1* is indicated by a red arrowhead.

Using the haplotypes called with 5-Mbp bins, we found that the median haplotype block length in wheat was 9.34 Mbp, containing a median of 196 genes. Blocks in centromeric regions (C) tended to be larger than those in distal regions (R1, R3)^8,15^ (C: 221.04 Mbp; R1: 15.43 Mbp, R3: 24.38 Mbp; *p* < 2e−16)(Fig. 2a, Supplementary Fig. 4). This size differential is consistent with the reduced recombination rates in peri-centromeric regions^16^. Given that gene density decreases towards centromeric regions^8^, we investigated whether these large blocks are relevant for breeding in terms of the number of genes they contain. Blocks that spanned centromeric regions carried more genes (on average) than distal blocks (C: 2,601 genes; R1: 467, R3: 623; *p* < 2e−16). These values illustrate that, regardless of chromosomal position, haplotype blocks fix sequence across regions containing hundreds if not thousands of genes, which is particularly relevant considering that these blocks are the effective units that are shuffled and assembled during breeding.

We compared the extent to which the 15 cultivars shared common haplotypes and observed that on average, 59.3 ± 4.6% of the genome was shared with at least one other cultivar (Fig. 2b). This value increased to 65.6 ± 1.8% when pre-1950 lines (Chinese Spring, Norin61) were excluded, consistent with modern cultivars being more highly related to each other than to the landraces. Common haplotypes were not equally distributed across chromosomes; for example, chromosome 6A of Jagger was almost completely shared with that of other cultivars (93.8%), whereas chromosome 4A of Jagger only shared 16.6% of sequence with other cultivars. The majority of common haplotypes (54.9 ± 3.2% of all haplotype blocks) were shared with at least two additional cultivars (i.e. present in at least three cultivars; Jagger is shown as a representative example in Fig. 2b), often with cultivars from different continents (Supplementary Fig. 5). In some cases, cultivars that shared a large number of common haplotype blocks are known to share common parents in their recent pedigree (e.g. Stanley and Landmark, 32.3% genome shared; Cadenza and Paragon, 38.8% genome shared). However, in many instances, cultivars with a high number of common haplotype blocks had no common parent within their documented pedigree (e.g. Robigus and Claire, 31.4% genome shared) (Supplementary Fig. 5; Supplementary Data 3). The high number of common haplotypes across modern cultivars reflects the relatively narrow genetic base of modern wheat post-Green Revolution and the extensive inter-crossing of germplasm across breeding programmes worldwide (Supplementary Fig. 2b).

We analysed the physical positions of the haplotype blocks across all cultivars to identify conserved regions that constitute targets for introgression of genetic variation. We identified regions that we defined as ‘highly conserved’ based on the sharing of a common haplotype with five or more other cultivars (6.1 ± 0.9% of the genome across all wheat chromosomes, Fig. 2c, Supplementary Fig. 6). These highly conserved regions likely represent haplotypes associated with breeding progress, such as the semi-dwarfing *RHT-B1b* haplotype on chromosome 4B (Fig. 2c, Supplementary Fig. 2c), and/or regions of overall low genetic diversity within the modern wheat gene pool. Defining these regions (Supplementary Data 4) will help prioritise research to establish the biological functions of these putative breeder-selected sequences through functional analyses (e.g. mutants, genome editing^17,18^). Similarly, the identification of these regions will allow the focused discovery of alternative haplotypes and the implementation of breeding strategies for the targeted introduction of genetic diversity with improved or novel functions.

### Using haplotypes for crop improvement

**Fig. 3:**
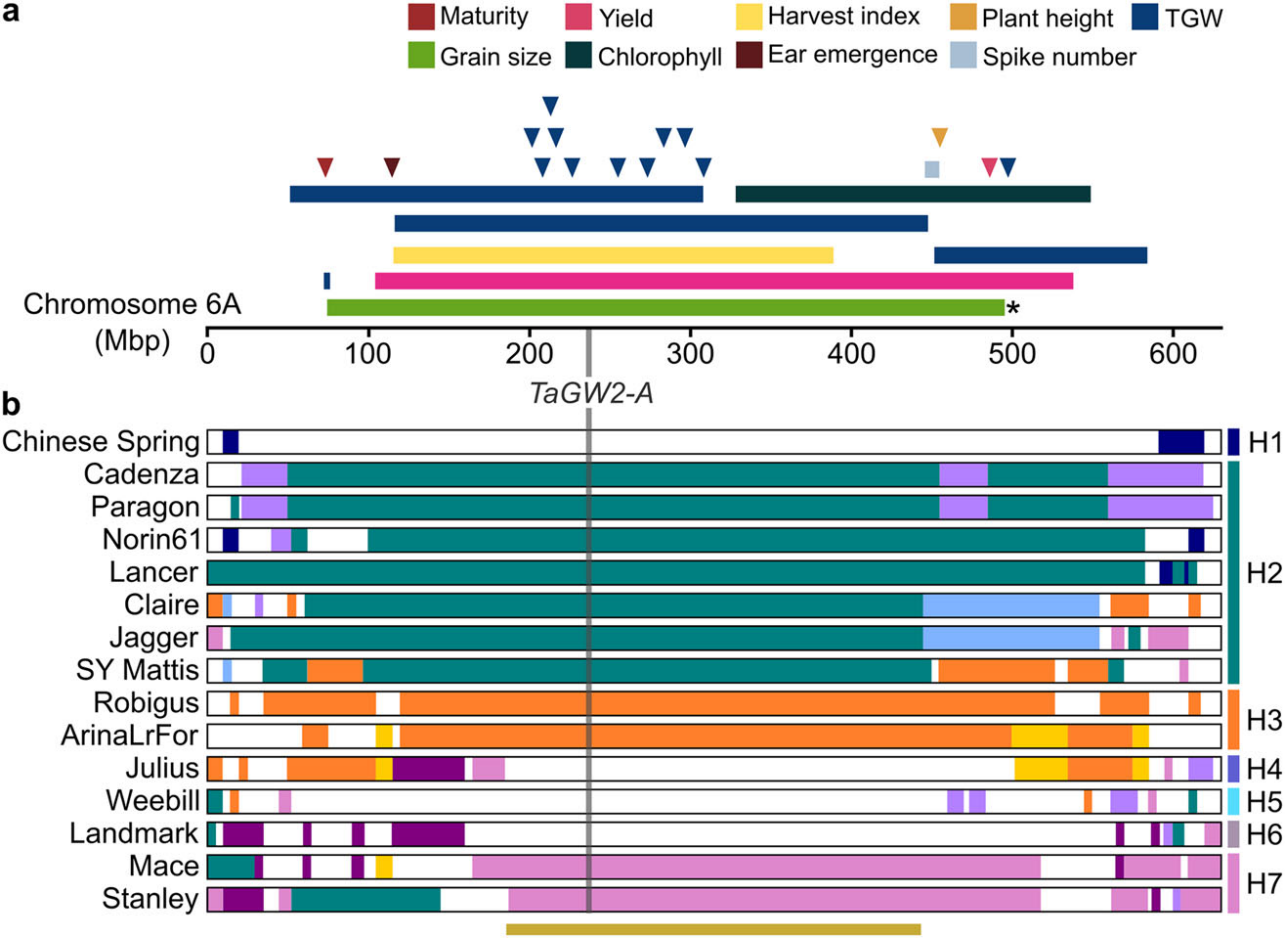
Haplotypes across the ‘highly conserved’ region of chromosome 6A. **a** Physical positions of productivity-related QTL (rectangles) and GWAS hits (triangles) mapped to the highly conserved region on chromosome 6A (see “Methods”). *; grain-size mapping interval based on UK cultivars Spark and Rialto. **b** Diagrammatic representation of all haplotype blocks on chromosome 6A in the 15 sequenced cultivars (based on 5-Mbp bin haplotypes; scaled to the longest chromosome 6A). Regions with the same colour at the same position share common haplotypes (except for white regions which are not contained within haplotype blocks). Vertical grey line indicates the position of *TaGW2-A* (237 Mbp). Labels H1–H7 indicate haplotype groups based on the minimum haplotype block (beige bar; 187–445 Mbp).

To demonstrate how our haplotype-based approach can support crop improvement, we focused on a ‘highly conserved’ region on chromosome 6A identified in the above analysis, and that is associated with productivity traits (e.g. yield, grain size, height, Fig. 3a, Supplementary Data 5). A single gene, *TaGW2-A*, is thought to be the underlying causal factor for yield-related traits based on associations with two linked SNPs in its promoter^19^. However, even though *TaGW2-A* was shown to regulate grain size using mutational approaches^20^, evidence for the functions of SNPs within its promoter is lacking, with conflicting results as to the beneficial allele^19,21^. We identified seven different haplotypes surrounding *TaGW2-A* amongst the 15 cultivars (haplotypes H1–H7, Fig. 3b), with H2 being shared across seven cultivars originating from diverse breeding programmes (UK, France, Japan, USA and Australia)^7^. Importantly, the *TaGW2-A* markers were unable to distinguish all seven haplotypes. This finding illustrates that selection/associations with single markers are often not diagnostic of the surrounding physical regions and highlights the caution required when selecting candidate genes based on single marker associations.

We further investigated the common haplotypes across chromosome 6A. In many cases, the haplotype blocks extended further and were larger than average, even when accounting for their centromeric location (genome C median: 221.04 Mbp; max 6A: 595.49 Mbp, Fig. 3b). We hypothesised that, in addition to suppressed peri-centromeric recombination, the conservation of these large blocks was due to local additive and/or epistatic effects where allelic combinations are maintained by breeders within the haplotypes. To address this hypothesis, we identified recombinant individuals in a cross between UK cultivars Spark (Haplotype H2) and Rialto (H3) in which we had previously mapped a grain-size QTL between 23 and 579 Mbp (Rialto provided the increasing effect in UK environments^22^) (Supplementary Fig. 7a). We identified 189 independent lines with recombination between 75 and 496 Mbp suggesting that despite the overall lower recombination frequency compared with the distal ends, these haplotypes can be recombined (Supplementary Fig. 7a–c, Supplementary Data 6). We evaluated a subset of the recombinant lines (38 lines in total) in multi-year field trials and observed an intermediate grain-size effect when the Rialto (H3) haplotype was disrupted through recombination between 75 and 496 Mbp (11 recombinant lines; Supplementary Fig. 7b, Supplementary Data 7). This result suggests that breeders have maintained multiple genes as an intact chromosome 6A haplotype to maximise phenotypic expression and have consequently limited genetic diversity across the chromosome.

**Fig. 4:**
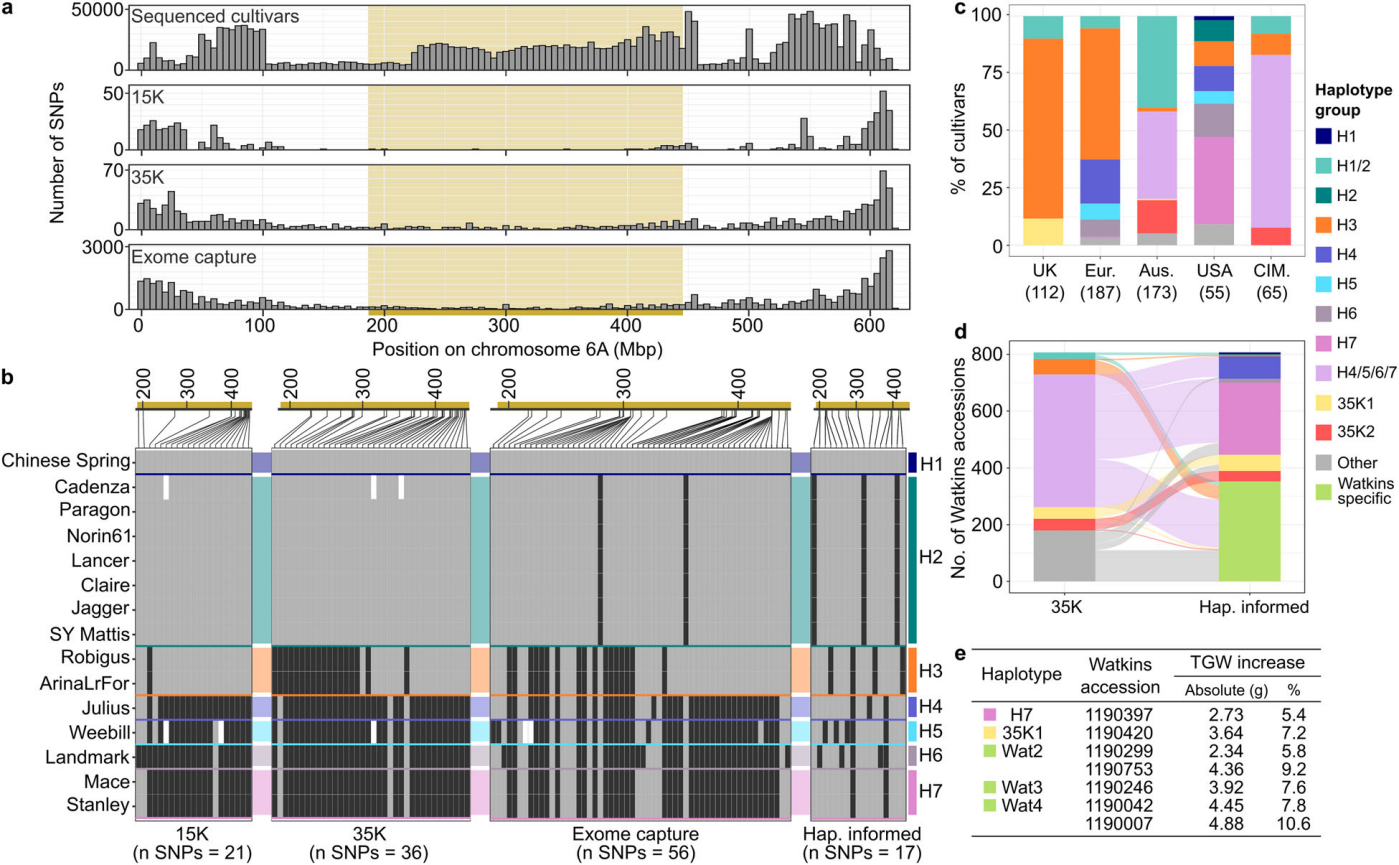
Identifying and exploiting novel haplotypes from the Watkins landraces. **a** Physical distribution of SNPs in 5-Mbp bins across chromosome 6A based on the 15 sequenced cultivars and other genotyping platforms (15K iSelect, 35K Axiom, exome capture). Beige shading indicates minimum haplotype block (MHB). **b** Allele calls extracted from sequenced cultivars for informative SNPs in MHB based on the genotyping platforms shown in (**a**) and allele calls for 6A haplotype-informed (Hap. informed) markers. Physical positions of SNPs are shown above each panel. Grey = reference allele, black = alternative allele. **c** 6A haplotypes present in germplasm panels based on genotyping platforms in (**b**) (number of cultivars in parentheses). ‘UK’ = UK Recommended List (35 K)^24^, ‘Eur.’ = European elite winter wheat (15K)^23^, ‘Aus.’ = Australian cultivars (35K)^24^, ‘USA’ = USA cultivars (exome capture)^11^, ‘CIM’ = CIMMYT Core Germplasm (CIMCOG) lines (35K)^24^. **d** 6A haplotypes in the Watkins landrace panel (*n* = 806) based on 35K array (left)^61^ and haplotype-informed markers (right). Full details of lines and haplotype allocations in Supplementary Data 10. Fill colour legend corresponds to both (**c**) and (**d**). **e** Summary of Paragon (H2) x Watkins bi-parental populations with QTL for increased thousand grain weight (TGW) across the MHB. See Supplementary Table 1 for additional phenotypes.

To further investigate the chromosome 6A region, we defined a 258 Mbp minimum haplotype block encompassing *TaGW2-A* and an additional 2167 genes for which there were no break points between haplotypes (Fig. 3b). To assess this region in a wider panel of global wheat cultivars, we leveraged public and novel genotyping data (*N* = 592 cultivars) originating from different platforms: 15K iSelect^23^ and 35K Axiom breeders’ array^24^ (SNPs derived from RNA-Seq), and exome-capture data^11^ (Supplementary Data 8). SNP density in all three platforms was skewed to the distal ends of chromosome 6A, which is consistent with their gene-based design (Fig. 4a). Consequently, there were relatively few markers within the 6A minimum haplotype block. This distribution was not reflected in the SNP density amongst assemblies, showing that markers in commonly used genotyping platforms do not capture the actual level of polymorphism between cultivars. We extracted the alleles of all informative markers from the 15 sequenced cultivars to assign genotyped panels to the previously defined haplotype groups. Although a handful of exome-capture SNPs were able to differentiate the seven haplotypes, neither the 15K nor the 35K breeders’ arrays provided sufficient resolution (Fig. 4b). Using the 15K array SNPs, we could not distinguish between H1 and H2, whilst with the 35K array SNPs we could only separate the sequenced cultivars into three discernible groups (H1/2, H3, H4/5/6/7). This lack of resolution is consistent with our finding that it is not possible to assign haplotype blocks accurately using only genic sequence (Fig. 1f).

Despite the limited resolution of breeders’ markers, we used the available array and exome capture data to conclude that H3 dominates in modern European germplasm and has been selected over time (χ2 = 13.6; df=1, *p* < 0.001), even though other haplotypes are present in the germplasm pool (Fig. 4c, Supplementary Fig. 8). We also identified, at low frequency (10.8 %), additional haplotypes that were not present in the 15 wheat genome assemblies (e.g. 35K1, 35K2). These findings suggest that the 15 sequenced cultivars capture a large proportion of modern global diversity across chromosome 6A and that cultivars carrying additional haplotypes could be targets for future sequencing efforts tailored towards particular environments (e.g. 35K1 in the UK, Fig. 4c). In the UK Recommended List cultivars, the dominant haplotype (H3) extended beyond the minimum haplotype block, encompassing approximately 68% of chromosome 6A (421.8 Mbp, 4,731 genes) (Supplementary Fig. 7d), suggesting that breeders selected an extended block due to local epistatic/additive effects of H3 in UK environments (Supplementary Fig. 7). By contrast, alternative haplotype blocks dominated in modern germplasm from Australia, USA and CIMMYT, despite the presence of H3 (Fig. 4c). It is likely that breeders selected different haplotypes across environments, possibly due to genotype*environment interactions^25^. For example, significant associations with TGW, yield and green canopy duration have been identified on chromosome 6A (Fig. 3a). In one study^25^, the phenotypic effects of a 6A haplotype was opposite depending on the environment in which it was tested (Mexico and Nepal). Likewise, the delayed final maturity associated with H3 (ref. ^22^) is likely to be selected favourably in environments with extended growing seasons (e.g. UK) compared to those with late-season heat (e.g. Australia).

Given the relatively low haplotype diversity across the 6A minimum haplotype block in modern germplasm, we tested whether we could use our haplotype approach to discover and introduce novel haplotypes from the Watkins landrace collection^26^ to increase diversity and potentially enhance agronomic performance. However, using the 35K data, most Watkins accessions were assigned ambiguously (H4/5/6/7; Fig. 4d). To assign haplotypes with better resolution, we designed a panel of haplotype-informed markers that distinguish all seven haplotypes from the sequenced cultivars and the additional haplotypes identified in the public datasets. As individual SNPs unique to a single haplotype were uncommon, we aimed to select an initial set of markers whereby each pairwise comparison between haplotype groups would have at least three differences in the marker profile with respect to every other haplotype to provide some redundancy. We also aimed to get a relatively even distribution of SNPs across the minimum haplotype block. After testing an initial set of 23 markers, we selected 17 markers that performed well as KASP assays to comprise the final set of haplotype-informed markers (Fig. 4b, Supplementary Data 9).

Using these haplotype-informed markers, we increased the haplotype resolution from 21 groups (16 Watkins-specific) to 40 (31 Watkins-specific) (Fig. 4d). All haplotypes present in modern cultivars (except H5) were identified in the Watkins landraces, suggesting that these large blocks have been maintained over time. Using the new haplotype-informed markers, we reassigned 243 lines previously assigned to modern haplotypes to Watkins-specific types, uncovering previously undetectable variation in this region (Fig. 4d, Supplementary Data 10). However, we confirmed that all UK Recommended List lines previously assigned to modern haplotypes remained the same when using the haplotype specific markers (Supplementary Data 10).

To identify Watkins-specific haplotypes with beneficial phenotypes, we used publicly available QTL data from Paragon (Haplotype H2) x Watkins bi-parental populations. Of the nine Watkins-specific haplotypes that were represented in bi-parental populations, we identified three associated with significantly increased grain weight (8.2 ± 0.8%, *p* < 0.05) (Fig. 4e, Supplementary Table 1). As these haplotypes are not present in modern germplasm, they represent novel variation that could be targeted for yield improvement in elite cultivars.

### Future perspective

The adoption of haplotype-led breeding will enable breeders to discover novel haplotypes and follow their introduction and phenotypic performance, as demonstrated for chromosome 6A. Defining haplotypes will promote more precise parental selection to maximise genetic gains within breeding programmes and enhance the ability to detect recombination events that partition and re-arrange haplotypes. This will allow breeders to evaluate local additive and epistatic effects^27,28^, to re-examine historic breeding trial data in a retrospective manner^1^, and to intentionally assemble optimised haplotype combinations that have directly predictable performance. This approach will be bolstered by gene editing to generate mutations within specific haplotypes^18^ and possibly targeted recombination to specified chromosomal regions^29^. To further facilitate the use of haplotypes in breeding and research, we developed an interactive visualisation platform (http://www.crop-haplotypes.com/) to investigate haplotype blocks in wheat. Our haplotype analyses provide a framework for exploiting natural diversity in a targeted, systematic manner with the aim to increase the precision of wheat breeding and optimise the agronomic performance of a key global crop.

## Methods

### Haplotype calls in wheat

To call haplotype blocks we used a combination of whole chromosome level NUCmer alignments and gene-based pairwise alignments using BLASTn^30^.

Nucmer alignments: We generated whole chromosome pairwise alignments between all 15 cultivars using NUCmer, excluding pairwise comparisons between scaffold-level assemblies. For the chromosome-level assemblies, we extracted individual chromosomes from the assemblies using samtools-1.9 faidx^31^. We performed pairwise alignments for each chromosome using the NUCmer program from MUMmer-3.23^32^ including the–mum option to use anchor matches that are unique in both the reference and query (this helps reduce the number of repeat induced matches). We filtered the raw delta files using the delta-filter command including the options -l 20,000 (minimum length of alignment 20,000 bp), -r and -q (as recommended for one-to-one mapping of reference to query—this leaves only alignments that form the longest consistent set for the reference and query). We used an -l of 20,000 to exclude shorter alignments between non-syntenic retrotransposons which have a median size of 9584 bp in wheat^9^. All reciprocal pairwise alignments between chromosome-level assemblies were performed and analysed. For alignments between chromosome-level and scaffold-level assemblies, we first filtered scaffolds to include only those that contained at least one RefSeqv1.1 gene model projection from the corresponding chromosome^7^. After scaffold filtering, we performed pairwise alignments and subsequent filtering as described above; in all cases the chromosome-level assembly was used as the reference and the scaffolds as the query. Pairwise alignments between scaffold-level assemblies were not conducted using NUCmer (see “BLAST alignments” below). To call haplotype blocks from the NUCmer alignments, we first calculated the percentage identity for each alignment and then binned alignments by position across the chromosome in 5-Mbp bins. It is important to note that the filtering for alignment size of ≥ 20,000 bp meant that the full 5-Mbp of sequence is not always contained within alignments. However, upon manual inspection of several regions we found that gaps in alignment sets were due to exclusion of smaller alignments between sequences disrupted by Ns and not shorter alignments of lower identity than the surrounding sequence. The median amount of sequence aligned in the 5-Mbp bins of chromosome-scale assemblies was 3,848,154 bp (i.e. 76.9% of bin breadth). Bins with a median sequence identity ≥99.99% were considered to be identical-by-state and therefore a shared haplotype. Whilst we would expect regions of identical-by-state to share 100% sequence similarity, the ≥99.99% allows for technical issues including the presence of Ns and sequencing errors, such as those that occur in areas of lower sequence complexity (Supplementary Fig. 1b; see “Methods”). Adjacent bins of the same haplotype were stitched together to obtain the coordinates of the haplotype blocks. When stitching blocks together, two consecutive ‘errors’ (bins < 99.99%) were allowed before splitting the blocks (see example in Supplementary Fig. 1a). Where reciprocal pairwise alignments were available (i.e. between chromosome-level assemblies) we only included haplotype blocks called in both reciprocal alignments in the final set. We also calculated blocks at lower levels of binning (2.5- and 1-Mbp) to provide increased resolution. The lower bin levels were calculated using the same criteria of ≥99.99% median and two consecutive errors.

BLASTn alignments: to complement the NUCmer alignments and allow for direct pairwise comparisons between scaffold-level assemblies, we performed pairwise BLASTn alignments of projected genes (GFF annotation RefSeqv1.1)^8^ ± 2000 bp for all pairwise comparisons between cultivars. For chromosome level assemblies, we retained only gene projections consistent with the expected chromosome. For the assemblies in scaffolds (Cadenza, Claire, Paragon, Robigus, and Weebill), we assigned each scaffold to a chromosome based on the origin of the projected genes. Then, we kept genes consistent with the expected chromosome. Finally, we filtered out genes with more than one projection in the expected chromosome. The script was written as a Jupyter notebook^33^ using BioRuby 2.0.1 (ref. ^34^), Daru 0.2.2 (ref. ^35^).

We wrote a Ruby^36^ script that takes the filtered genes and performed pairwise BLASTn alignments between all cultivars for each gene. From the XML output (-outfmt ‘5’), we extracted the largest HSP in the alignment, including the alignment length, percentage identity and number of Ns. We filtered out alignments containing Ns in the aligned sequence. For each pairwise comparison, the gene-based alignments were ordered based on the Chinese Spring RefSeqv1.0 physical position. We then called haplotype blocks using a sliding window of 25 consecutive genes. For each window of 25 genes, we ordered the gene-based alignments by percentage identity and removed the 10% of alignments with the lowest percentage identity (i.e. 3 lowest). We then calculated the mean of the percentage identity of the remaining alignments. If the mean was equal to 100%, we considered the window to be identical-by-state i.e. a shared haplotype. After calling blocks using windows based on the RefSeqv1.1 gene order, we converted the coordinates of each block to the corresponding assembly using the method described below (section “coordinate conversion between assemblies”). In addition to ± 2000 bp, we also performed pairwise alignments for the coding sequence (CDS), the gene including the introns (± 0 bp), ± 1000 bp and ± 5000kbp. We selected the parameters of ± 2000 bp flanking sequence and a 25 gene window based on a precision/recall analysis (described below, Supplementary Fig. 3a).

Combining NUCmer- and BLAST-based blocks: To generate the final set of genome-wide haplotype blocks we combined the blocks called from the NUCmer and BLAST approaches, removing any BLAST blocks already represented in the NUCmer set and smaller than the NUCmer bin size. In total we identified 4485 pairwise haplotype blocks genome wide using 5-Mbp bins, 7578 using 2.5-Mbp bins and 17,693 using 1-Mbp bins (Supplementary Fig. 3b, Supplementary Data 2).

Parameter selection for BLAST-based haplotype calling: To select parameters for the BLAST-based haplotype blocks, we tested a range of flanking sequence surrounding genes (CDS only and gDNA ± 0, 1000, 2000 or 5000 bp) and a range of window sizes (10, 15, 20, 25 and 30 genes). We then called blocks using all combinations of these parameters for a subset of chromosomes (6 A, 7 A, 2B, 3B, 1D and 4D). For each chromosome from each pairwise comparison of cultivars, we looked for overlaps between the previously called NUCmer blocks and the BLAST blocks called using the different combinations of parameters and calculated precision/recall scores (Supplementary Fig. 3a). Precision was calculated as the number of BLAST blocks overlapping with at least one NUCmer block divided by the total number of BLAST blocks. Recall was calculated as the number of NUCmer blocks overlapping with at least one BLAST block divided by the total number of NUCmer blocks. The F1 score was calculated as (2*((precision*recall)/(precision+recall))). We then combined results from all six chromosomes tested to select the final parameters. We found that precision increased with increasing flanking sequence whilst the recall decreased. We also found that loss of data due to Ns increased substantially with increasing flanking sequence, reducing the power to identify haplotype blocks (i.e. lower recall). We therefore selected 2000 bp flanking sequence, which provided a balance between accuracy and loss of data. Similarly, the precision increased with increasing window size whilst the recall decreased. Whilst the 20, 25 and 30 gene windows had similar F1 scores, the 25 gene window provided a balance between precision (> 20 gene window) and recall (> 30 gene window).

### Visualisation interface and coordinate conversion between assemblies

To visualise the haplotypes a Ruby on Rails^37^ was developed with a MySQL^38^ relational database storing the sequenced cultivars annotation and block annotation as an extension of the database described in ref. ^39^. To display the haplotypes in an intuitive way the website incorporates a novel visualisation developed in Javascript, with the D3js^40^ library. The source code and instructions on how to install the server is available (https://github.com/Uauy-Lab/pangenome-web).

In addition to the visualisation code, the database was used for the following analysis:

Coordinate conversion between assemblies: Coordinate conversion was performed using the projected RefSeqv1.1 genes as a common factor across all assemblies. The algorithm works as follows: first, we extract the projected genes within the physical interval to convert (i.e. in the haplotype block), along with the corresponding gene IDs and coordinates from the RefSeq v1.1 annotation. We then identify the projections of these genes in the target assembly with their mapped coordinates and order the genes by their position in the target assembly. We then join all adjacent genes in blocks, allowing gaps of up to 20 genes. If none of these blocks contains at least 10 genes, we keep the longest block, otherwise, we keep all the blocks with more than 10 genes. Finally, we extract the coordinates of the converted block in the target assembly based on the first and last coordinates within each block (Supplementary Fig. 9). The reciprocal algorithm is implemented to be able to convert from the sequenced cultivar assemblies to the RefSeq assembly. This algorithm is implemented in the programmes haplotypes:convert_gene_coordinates and haplotypes:convert_bed_coordinates, used to convert the haplotype blocks or arbitrary BED files.

Haplotype block length and gene content: The algorithm described above is implemented in the program haplotypes:export_haplotype_block_stats_in_points (see “Haplotype block length and gene content” for more details).

### Calculation of sequence complexity

To calculate sequence complexity surrounding SNPs we used the show-snps from MUMmer to extract all SNPs plus 10 bp flanking sequence from all pairwise NUCmer alignments of chromosomes 2B and 6A (using -C option to exclude SNPs with ambiguous mapping). We excluded sequences with Ns. We calculated the Shannon richness of k-mers of SNP surrounding sequence using seqComplexity() from the R library dada2 (ref. ^41^) with a kmerSize of 2. In all cases we used the reference sequence from each pairwise comparison. For each pairwise comparison, we assigned SNPs as either within a haplotype block in the specific pairwise comparison, or not, according to the 5-Mbp haplotype blocks called using NUCmer alignments. We performed pairwise Wilcox tests with Benjamini-Hochberg adjustment^42^ for multiple testing in R to test differences in sequence complexity between SNPs inside and outside haplotype blocks. We performed tests for the total set of SNPs in each pairwise comparison, in addition to subsampling 10,000 SNPs from each category where possible (i.e. when haplotype blocks were long enough to include 10,000 SNPs) from each pairwise comparison. We also calculated significance for the combined sets of subsampled SNPs across all pairwise comparisons for each chromosome.

### *RHT* analyses and expected sequence divergence

For the 15 wheat cultivars, we identified *RHT-B1* (Traes4B02G043100) and *RHT-D1* (TraesCS4D02G040400) alleles^43^. Using ~300 Kbp of sequence surrounding each gene, we performed a BLASTn alignment for a representative cultivar with the GA-insensitive *RHT-B1b* allele (Jagger) against the GA-sensitive alleles *RHT-B1a_1* (CS), *RHT-B1a_2* (Arina), *RHT-B1a_4* (Julius) and *RHT-B1a_8* (Mace). A similar comparison was done for the D genome GA-insensitive *RHT-D1b* allele (Mace) against the GA-sensitive alleles *RHT-D1a_1* (CS), *RHT-D1a_2* (Arina) and *RHT-D1a_3* (Jagger). For each pairwise comparison, the total sequence used and the number of Ns per sequence was determined to calculate the ‘maximum alignable’ sequence (total sequence minus Ns). SNPs and small indels, alongside matched sequences were tallied for each BLAST alignment, and the ‘total aligned sequence’ was calculated (SNPs/indels plus matched sequence). The percentage of sequence identity was calculated by dividing the total matched sequence by the total aligned sequence. The sequence identity values calculated from the BLASTn alignment of the ~300 Kbp were consistent with the values obtained in the tabulated NUCmer output files which only include alignments ≥20 Kbp. The breadth of the total alignment was calculated by dividing the ‘total aligned sequence’ by the ‘maximum alignable’ sequence. The average breadth of the total alignment in the 7 pairwise comparisons (*RHT-B1b* and four *RHT-B1a* alleles and *RHT-D1b* and three *RHT-D1a* alleles) was 99.6 ± 0.2%, i.e. an average of 297,244 bp aligned out of the possible 298,315 bp queried. Indels which disrupted the BLAST alignment were included in the comments section of Supplementary Fig. 2a. This 99.6% breadth contrasted with the NUCmer output files which aligned 88.5 ± 2.3% (4 *Rht-B1* comparisons) and 77.4 ± 5.4% (3 *Rht-D1* comparison) of the ~300 Kbp sequence. This was due to alignments shorter than 20 Kbp which were considered in the analysis above but that were filtered in the NUCmer output (-l 20000).

Cultivar pedigrees and *RHT* allelic status were based on http://wheatpedigree.net/ and published work^44^, and were visualised using Helium^45^ (Supplementary Fig. 2b). The size of haplotype blocks for *RHT-B1b* and *RHT-D1b* were identified in Supplementary Data 2 and precise breakpoints defined through manual sequence comparison across these regions. These were used to represent the region diagrammatically and to scale, alongside a screenshot of the visualisation viewer (Supplementary Fig. 2c). To calculate the expected sequence identity based on 10,000 years divergence we used the nucleotide substitution rate for repetitive/intergenic DNA in wheat which was estimated to be 1.6 ×10^–8^ nt^−1^ year^−1^ (ref. ^10^).

### Haplotype block length and gene content

To calculate the median length and gene content of haplotype blocks, we first converted haplotype block coordinates to the RefSeqv1.0 coordinate system (as described above). This enabled us to calculate gene content using the RefSeqv1.1 high-confidence (HC) and low-confidence (LC) genes, in addition to placing the haplotypes into chromosomal compartments as calculated based on recombination for Chinese Spring^8,15^. For the whole genome block length and gene content we calculated median values including all pairwise haplotype blocks. To calculate block length and gene content in relation to chromosome position (data shown in Fig. 2a, Supplementary Fig. 4a), we sampled each chromosome at fixed positions every 500-Kbp and then scaled the physical positions to a percentage of the chromosome length ((position of sampling point/chromosome length)*100). We assigned blocks to a 500 Kbp position if they intersected. Hence a 5-Mbp block would be considered at multiple 500 Kbp sampling positions (between 10 and 12 depending on exact start and end position of the 5-Mb block). We used this sampling approach to accommodate the fact that many blocks span multiple chromosomal compartments. We calculated median values of sampling positions in 1% bins across each chromosome and then combined these values to calculate distributions of 1% bins summarised across all chromosomes. We then assigned the 1% bins to chromosomal compartments (R1, R2a, C, R2b, R3) and used the median values calculated for each chromosome to calculate the medians for each compartment. The minimum and maximum values for each compartment were calculated based on the raw values, not on the chromosome summarised values (Supplementary Fig. 4b).

### Haplotype sharing between cultivars

To calculate the percentage of the genome/individual chromosomes shared between pairs of cultivars (Supplementary Fig. 5), we first took all haplotype blocks identified in each pairwise comparison. We then calculated the total physical size of blocks as a percentage of the chromosome/genome size of the reference cultivar in question.

To assess shared haplotypes across all cultivars, we loaded all block coordinates as a GenomicRanges^46^ object in R. For each cultivar, we used the coverage function to calculate the number of other cultivars that shared a common pairwise haplotype block with the cultivar in question at any given position. For the percentage of genome/chromosomes contained within haplotype blocks across all cultivars (Fig. 2b), we calculated the total amount of sequence contained within at least one haplotype block for each cultivar (i.e. coverage ≥1) and expressed this as a percentage of the genome/chromosome size based on the reference cultivar in question. We then used the coverage value to assess how conserved regions were across cultivars. We classified regions with coverage ≥5 to be ‘highly conserved’ i.e. the haplotype was shared amongst at least 6 cultivars (reference + 5 others) (Supplementary Data 4). We then scaled coordinates to the percentage of the total chromosome length with respect to the reference cultivar used so that we could compare conserved regions across cultivars (as shown in Fig. 2c and Supplementary Fig. 6). Supplementary Fig. 6 includes the positions of genes of agronomic importance based on published studies^7,8,47,48^.

### Assigning previously identified QTL to physical positions

We performed a literature search for studies which had previously identified associations with productivity-related traits in the ‘highly conserved’ region on chromosome 6A. We identified associations with grain size^22^, thousand grain weight (TGW^22,25,49–57^), yield^25^, spike number^50^, plant height^53^, harvest index^58^, chlorophyll^50^, ear emergence^59^ and maturity^53^. To assign physical positions we used the probe sequence (where available) for the peak (GWAS) or flanking markers (QTL) as queries in BLASTn alignments against the IWGSC RefSeqv1.0 assembly. In cases where only primer sequences were available for markers, we required that both markers had a BLAST hit in the same region on chromosome 6A (exact distance tolerated depending on the type of marker). All alignments were manually inspected. Data are presented in Supplementary Data 5.

### Germplasm, DNA extraction and KASP genotyping

Seeds for the Watkins landrace collection was obtained from the John Innes Centre (JIC) Germplasm Resources Unit (GRU). Seed for the UK Recommended List cultivars was also obtained from the JIC GRU; if not available it was obtained directly from the companies which bred the cultivars (RAGT, KWS, Elsoms, Limagrain or Syngenta). DNA extraction of young leaf samples and KASP genotyping were conducted as previously described^60^. 35K Axiom genotyping for the 111 Recommended List cultivars (Fig. 4b) was performed at the Bristol Genomics Facility using established protocols^24,61^.

### 6A recombinants and field trials

An initial set of 6A recombinant inbred lines (RILs) were developed by screening 212 BC_4_F_2_ plants for recombination between markers *gwm334* and *gwm570* (ref. ^22^). 67 recombinants were identified and self-pollinated to generate homozygous BC_4_F_3_ RILs. We defined the recombination events using 40 additional KASP markers (Supplementary Data 6) and found 11 independent RILs with recombination within the ~70–500 Mbp interval to which we mapped the grain width phenotype (Supplementary Fig. 7). We assigned the parental lines, Spark and Rialto, to haplotype groups H2 and H3, respectively, based on 35K Axiom breeders’ array data (see below).

These 11 RILs were evaluated at the JIC Experimental Field Station in Norwich (52.628 N, 1.171 E) in five trials across four years: large-scale yield plots (1.1 x 6 m) in 2013–2016 and an additional trial of 1.1 x 1 m plots in 2015. Exact lines grown in each trial are detailed in Supplementary Data 7. In all five trials a randomised complete block design was used with at least five replications. Grain width was recorded on the MARVIN grain analyser (GTA Sensorik GmbH, Germany) using ~400 grains obtained from combine-harvested grain samples.

We subsequently conducted a second, larger screen (2674 BC_4_F_2_ plants) with the aim of further documenting recombination events across the extended chromosome 6A haplotype block. We identified 428 independent heterozygous RILs between *BS00066522* and *BS00066623* and confirmed 178 independent homozygous BC_4_F_3_ RILs after self-pollination. Note that not all heterozygous BC_4_F_2_ RILs were taken forward to the BC_4_F_3_ generation. We further defined the location of recombination events in the homozygous RILs using 5 additional markers (*BS00003881* (83,259,238 bp)*, BS00185740* (105,022,477 bp)*, BS00041481* (176,574,306 bp)*, Hap-P2 (TaGW2-A*; 237,734,146 bp) and *BS00009988* (352,615,648 bp); Supplementary Fig. 7c). These results support the idea that these haplotypes can be recombined, although given the more recent generation of these additional RILs we did not evaluate them phenotypically in multi-year field trials.

### SNP data analysis

15K iSelect: We downloaded the genotype matrix and physical positions of SNP markers (Supplementary Data 1 from ref. ^23^) and extracted all markers located on chromosome 6A according to the physical positions. To assign genotyped lines to 6 A haplotype groups H1–7, we extracted markers within the minimum haplotype block (MHB) based on the physical positions provided (187–445 Mbp; defined as the region across which there was no recombination between haplotypes in the 15 sequenced cultivars (Fig. 3b)). We assigned lines to haplotype groups based on having the same marker profile across the MHB as the sequenced cultivars (see below for details about allele identification in sequenced cultivars). We identified one marker profile that was not represented in sequenced cultivars.

35K Axiom: We downloaded the Breeders’ 35K Axiom genotyping data from cerealsdb.uk.net^62^ along with physical positions according to RefSeqv1.0 and genetic map information. We extracted all markers located on chromosome 6A based on having both a RefSeqv1.0 position on 6A and a consensus genetic map position on 6A. To assign genotyped lines to 6A haplotype groups H1–7, we filtered to include only markers that had calls for both alleles in the whole dataset (total 2,188 lines) and then extracted markers in the MHB based on physical positions (187–445 Mbp). We assigned haplotype groups based on marker profiles across the MHB. For haplotype assignment, we excluded AX-95201760 as it had inconsistent allele calls amongst repeated lines, and also between Cadenza samples and the Cadenza assembly. We also excluded AX-94532884, AX-94560723 and AX-94531101 as the probe sequences did not have unambiguous BLAST hits in at least four of the sequenced cultivars, and therefore alleles could not be extracted (see below for details). In this study, we present the genotypes from Australia, CIMMYT Core Germplasm (CIMCOG), UK Recommended List and the Watkins landrace collection^61^. We also generated 35K Axiom data for an additional 111 lines from the UK Recommended List and used the same set of markers across the MHB as described above to assign lines to 6A haplotype groups (Fig. 4c,d; Supplementary Data 8, Supplementary Fig. 7).

Exome capture: We downloaded the processed VCF (after imputation and filtering) of SNPs called for 811 accessions^11^. SNPs were called according to the IWGSC RefSeqv1.0 so we therefore used these physical positions. We extracted the genotypes for SNPs on chromosome 6A, filtering for those located in regions covered by IWGSC RefSeqv1.1 HC + LC genes using vcftools (start to end of gene including introns and UTRs where annotated). We included this quality control given that the SNPs originated from an exome capture (i.e. SNPS expected in exons and adjacent intron sequence) and that the VCF files do not include information about quality/depth of SNPs. Lines were assigned to 6A haplotype groups based on the marker profile across the MHB (187–445 Mbp). Here, we present the haplotype allocations for the cultivars from the USA.

Extracting alleles of markers and exome capture SNPs from sequence cultivars: To identify alleles for all markers in the MHB for each platform, we performed BLASTn alignments against all 15 genome assemblies using the SNP context sequence as the query (-perc_identity 98 -max_target_seqs 1). For the 15K iSelect and 35K Axiom arrays we used the probe sequence and for the exome capture we extracted 100 bp up and downstream of the SNP position from the IWGSC RefSeqv1.0 assembly. We filtered the BLAST output to retain only those results with full length hits to chromosome 6A (in the case of chromosome level assemblies) and extracted the allele from the corresponding location in the BLAST query sequence. For marker-assembly combinations for which a full length hit to chromosome 6A was not found, BLAST alignments were manually inspected to identify the correct allele call. In the case of scaffold level assemblies, manual inspection of BLAST hits was used to ensure scaffolds originated from chromosome 6A based on homoeologous SNPs observed in chromosome level assemblies. Only sequences which could unambiguously be assigned to chromosome 6A based on the BLAST alignment were retained and given an allele call.

Identification of SNPs on chromosome 6A across 15 sequenced cultivars: We used show-snps from MUMmer to extract SNPs from pairwise alignments of chromosome 6A between Chinese Spring and all other cultivars (using -C option to exclude SNPs with ambiguous mapping). We removed SNPs where SUB1 or SUB2 (i.e. reference and query alleles) were either “N” or “.”. We then collated SNPs from all comparisons and removed duplicate reference SNP positions to get the final set of chromosome 6A SNPs summarised across all 15 sequenced cultivars with respect to the Chinese Spring assembly.

### Development of 6A haplotype markers

To choose a panel of SNP markers that could distinguish haplotypes across the 6A MHB (H1-H7, plus additional haplotypes identified in the wider germplasm panels (e.g. haplotypes 35K1, 35K2)), we considered a combination of markers from the 15K, 35K and exome capture in addition to novel SNPs identified amongst the 15 sequenced cultivars. We aimed to include 15K and 35K arrays if informative as these markers are already used by breeders and therefore may reduce the number of additional markers required for breeders to assign material to haplotype groups. We developed KASP assays using Polymarker^63^ for 23 SNPs across the MHB, which we tested on DNA samples of the 15 sequenced cultivars. Of these, 17 markers performed well (e.g. distinct clusters) and were selected as the final set of haplotype-specific markers (Supplementary Data 9). We ran the 17 KASP markers on DNA from a panel of 1,011 Watkins landraces and a panel of 85 UK Recommended List lines. Whilst 243 Watkins landraces previously assigned to modern haplotypes (H1-H7, 35K1, 35K2) were reassigned to Watkins-specific haplotypes using these markers, all UK Recommended List lines previously assigned to modern haplotypes remained the same when using the haplotype-specific markers (Supplementary Data 10).

### Watkins lines field phenotyping and QTL analysis

The Paragon x Watkins bi-parental mapping populations were evaluated at the John Innes Centre Experimental Field Station (Bawburgh, Norfolk; 52.628 N, 1.171 E) in 2011, 2013, 2014, 2015, 2016 and 2017 (Fig. 4e, Supplementary Table 1). Initial crosses of Paragon (female) to Watkins landrace (male) plants were advanced to F_4_ using single seed descent. Seed source for plots were F_4:5_ seeds derived from a single F_4_ plant used for DNA extraction and genetic map development^26^. Populations were drilled in autumn (September/October) in all years as 1 m x 1.5 m plots (three rows spaced at 0.5 m). Heights were recorded by hand from the centres of the plots at maturity. All grain characteristic (grain weight, length, width, and surface area) were determined on the MARVIN grain analyser (GTA Sensorik GmbH, Germany) with ~400 grains from harvested plots.

Genetic maps were constructed as described^26^. QTL mapping was conducted in the R software suite (v3.6.1) using package “qtl” (v1.41)^64^. The analysis took cross type and generation number of the populations into account by using “read.cross” options “BC.gen = 0” and “F.gen = 4” or “F.gen = 5”. The QTL model used a significant threshold calculated from the data distribution. A first QTL scan, using Haley-Knott regression, determined co-factors for the second scan. The second scan by composite interval mapping (CIM) identified QTL at a significance level of 0.05 taking the co-factors into account. All data can be downloaded from http://wisplandracepillar.jic.ac.uk/results_resources.htm.

### Statistics and reproducibility

Data are presented as mean ± s.e.m. For all boxplots, the box represents the middle 50% of data with the borders of the box representing the 25th and 75th percentile. The horizonal line in the middle of the box represents the median. Whiskers represent the minimum and maximum values, unless a point exceeds 1.5 times the inter-quartile range in which case the whisker represents this value and values beyond this are plotted as single points (outliers). In Fig. 2a and Supplementary Fig. 4a, outliers are not plotted. The chromosome 6A RILs were evaluated using two-way ANOVAs with the model including the trial and genotype as factors. When RIL groups were assessed, independent RILs within each group were considered as replicates within the model. RILs/RIL groups were assigned to parental genotypes using a post hoc Dunnett’s test to compare with both the S- and R-controls. RILs significantly similar to the S-control and significantly different from the R-control were classified as S, whereas RILs indicates significantly similar to the R-control and significantly different from the S-control were classified as R. RILs classed as not statistically different from both the S and R control groups were classed as intermediate. All measurements were taken from independent experimental units (biological replicates). Statistical analyses were performed using Minitab Statistical Software. We conducted a CHI^2^ test of independence (‘MASS’ library^65^) to compare the frequency of haplotype H3 to all other haplotypes in the European germplasm based on year of release (pre-2000 vs. 2000 onwards). Pairwise Wilcox tests with Benjamini–Hochberg adjustment for multiple testing were conducted for sequence complexity analyses and for comparisons of block length and gene numbers across chromosomal compartments.

## Supplementary information

Supplementary information

Description of Additional Supplementary Files

Supplementary data 1

Supplementary data 2

Supplementary data 3

Supplementary data 4

Supplementary data 5

Supplementary data 6

Supplementary data 7

Supplementary data 8

Supplementary data 9

Supplementary data 10

## Data Availability

All sequence reads and assemblies for the five scaffold-level assemblies are deposited at the SRA under accession PRJEB35709, whereas the chromosome-scale reads are deposited as accession PRJNA544491 and assemblies available at https://wheat.ipk-gatersleben.de/. Haplotype visualisation is available at http://www.crop-haplotypes.com/. Seed stocks of the assembled cultivars and Watkins landraces are available at the UK Germplasm Resources Unit (https://www.seedstor.ac.uk/). Watkins phenotypic and QTL data are available at http://wisplandracepillar.jic.ac.uk/results_resources.htm (accessed April 2020). NUCmer output and the filtered tabulated rds files for all alignments, output from all pairwise BLASTn alignments of projected genes ± flanking sequence and all the files to setup the haplotype visualisation website are deposited in https://grassroots.tools. Final haplotype blocks are available as Supplementary Data 2 using 5000, 2500 and 1000 Mbp windows. Markers and grain width data for 6A recombinant across five trials is available as Supplementary Data 6 and 7. Genotyping calls across all panels are available as Supplementary Data 8 to 10. There are no restrictions on data availability, and we encourage data reuse.

## Code availability

All code used for data analyses and scripts for plots in main and Supplementary Figures are deposited in https://github.com/Uauy-Lab/pangenome-haplotypes. Specific scripts include: nucmer_all_chromosomes_chromosome_level_assemblies_wheat.sh: Generation and filtering of NUCmer raw delta files. nucmer_all_chromosomes_scaffold_level_assemblies_wheat.sh: Pairwise alignments of chromosome-level and scaffold level assemblies, and subsequent filtering of NUCmer raw delta files. assign_mummer_blocks_whole_genome.r: Defining haplotype blocks from NUCmer alignments. assign_BLAST_blocks_whole_genome.r: Assignment of haplotype blocks using a sliding window of 25 consecutive genes. combine_mummer_and_BLAST.r: Combining the blocks called from the NUCmer and BLAST approaches. Filter projected genes.md: Filter projection genes based on chromosome. The code for the pairwise-blast is released as a Ruby Gem (bio-pangenome v 0.1.3) and the source code is in https://github.com/Uauy-Lab/bioruby-pangenome. slice_block_stats.sh: Extract the stats of how many genes intersect each 500 kbp sampling point. convert_gene_blocks.sh: Convert the haplotype blocks from the pairwise blast to the corresponding genomes. regions-without-IBD.Rmd: Calculates the number of haplotypes shared across lines. calculate_BLAST_blocks_all_window_sizes_flanking_sequences.r: Assignment of haplotype blocks using sliding windows of 10, 15, 20, 25, 30 consecutive genes with a range of flanking sequences (CDS, ± 0, 1000, 2000, 5000 bp). calculate_combined_precision_recall.r: Calculates the precision, recall and f1 scores between blocks called using NUCmer and BLASTn. mummer_show_snps_all_aln_per_chr.sh: Extracts all SNPs and 10 bp flanking sequence called in pairwise NUCmer alignments for a given chromosome calculate_sequence_complexity.r: Calculates sequence complexity from mummer_show_snps_all_aln_per_chr.sh output. calculate_seq_complexity_stats.rmd: Subsamples snps from pairwise comparisons and performs pairwise wilcox texts of sequence complexity between snps inside and outside haplotype blocks. calculate_snp_distribution_6A_CS.r: Calculates SNP distribution across 6A with respect to Chinese Spring based on pairwise NUCmer alignments using output from show_snps
