# Supplementary material for: A haplotype-led approach to increase the precision of wheat breeding
Source: Commun Biol. 2020 Nov 25;3:712. doi: 10.1038/s42003-020-01413-2 (PMC7689427; doi:10.1038/s42003-020-01413-2)
Supplement: Supplementary file 1 — Supplementary information [file 42003_2020_1413_MOESM1_ESM.docx]

# Supplementary Information


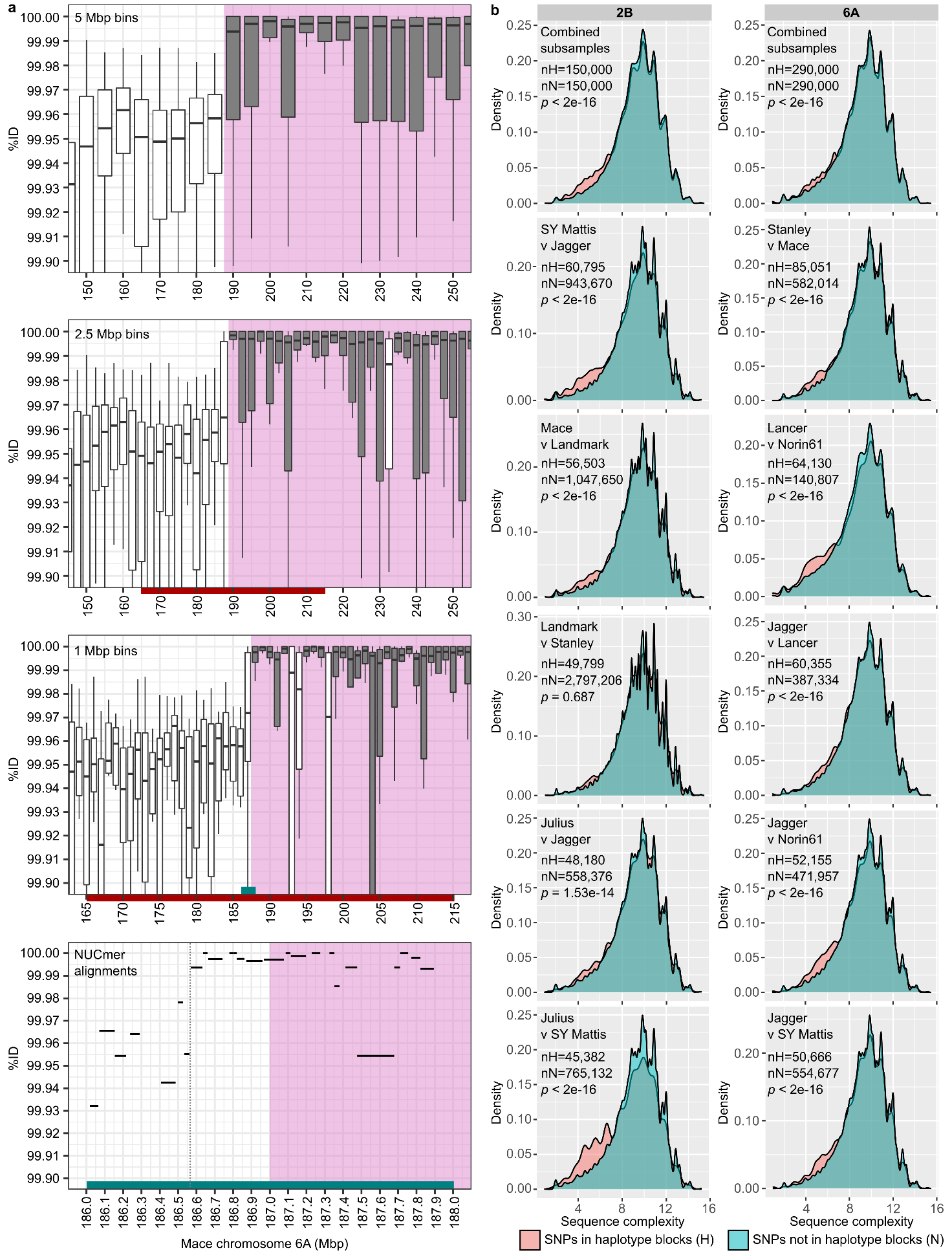


## Supplementary Fig. 1 | Comparison of near-identical sequences and haplotype blocks. a, Close up of transition zone between near-identical sequence (white, ≤ 99.99% sequence identity) and region called as a haplotype block using NUCmer (pink-shaded region). Boxplots are data as in Fig. 1d in 5-, 2.5- and 1-Mbp bins (as indicated in each panel). Grey-filled boxplots indicate bin median ≥ 99.99% sequence identity. Red and teal bars on X-axis indicate zoom of region between the 2.5- and 1-Mbp bin panels and the 1 Mbp and alignment panel, respectively. Bottom panel shows the % sequence identity of NUCmer alignments ≥ 20 Kbp with respect to Mace physical position. Note that the haplotype block (pink-shaded region) starts at 187 Mbp despite the alignments suggesting that the start of the block is at 186.6 Mbp (dashed vertical line in Alignments panel). This is due to the bin size, which determines the fine-scale resolution. b, Sequence complexity for ± 10 bp sequence surrounding SNPs within haplotype blocks (H, red) and outside haplotype blocks (N, teal) across chromosome 2B and 6A. Top panel shows the combined values from subsampling 10,000 SNPs (where possible) from within and outside haplotype blocks from all pairwise comparisons. The bottom panels show five specific examples for each chromosome of the individual distributions of pairwise SNPs for the five comparisons between cultivars with the highest number of SNPs in haplotype blocks, excluding reciprocal comparisons. nH and nN indicate the number of SNPs found within and outside haplotype blocks, respectively, for each comparison. P values correspond to pairwise Wilcox tests with Benjamini-Hochberg adjustment for multiple testing.


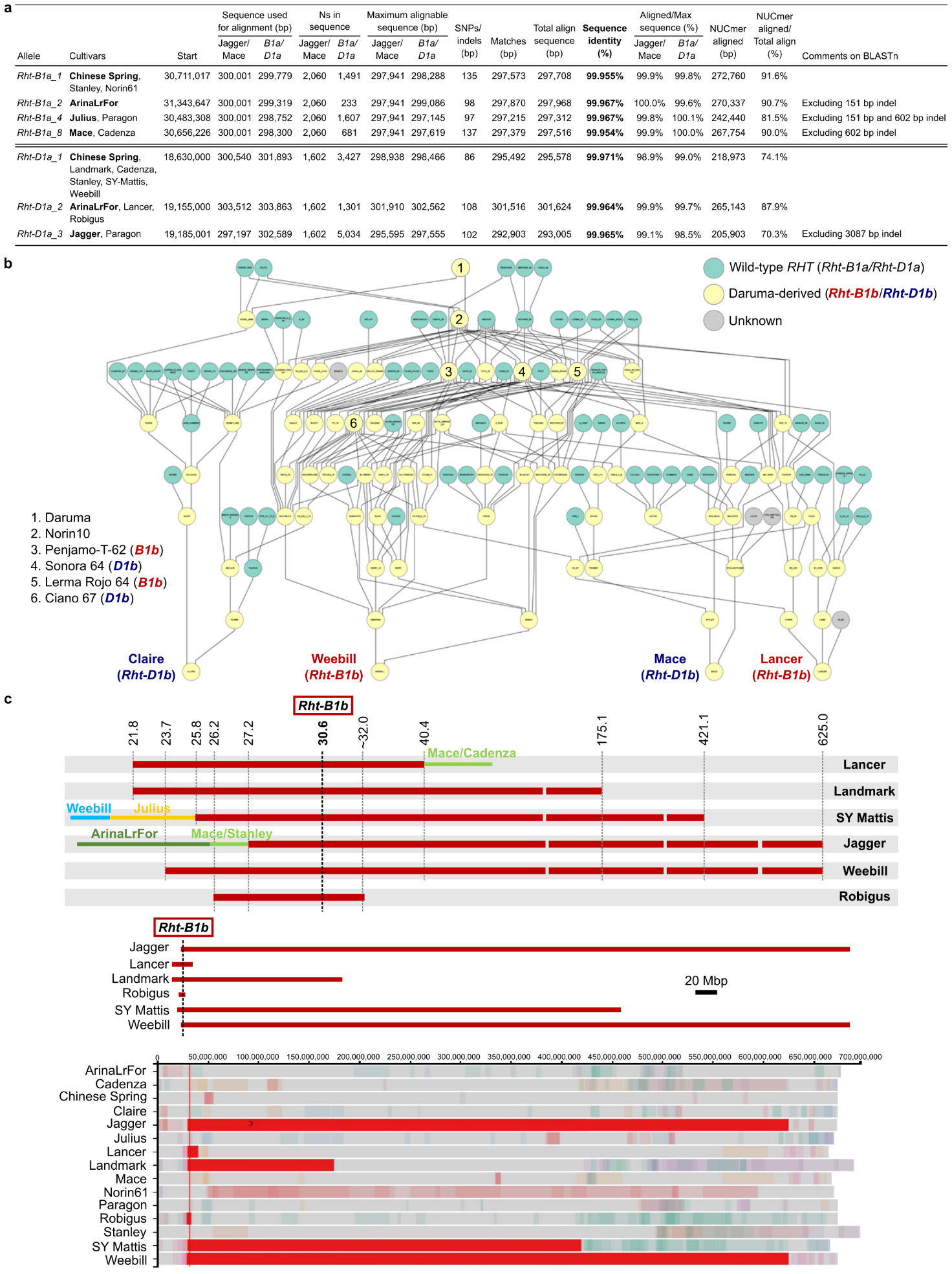


## Supplementary Fig. 2 | Analysis of *RHT-B1* and *RHT-D1* sequences, pedigrees and haplotypes. a, Comparison between ~300 Kbp sequence surrounding *RHT-B1* and *RHT-D1* in sequenced cultivars. Top section of the table shows the comparison between different *RHT-B1a* alleles (representative cultivar in bold) and *RHT-B1b* (Jagger), whereas bottom of table shows similar comparisons but for *RHT-D1a* and *RHT-D1b* (Mace). The table indicates the sequences used for BLASTn alignments, number of Ns in each sequence and the maximum sequence (total minus Ns). Total matches and SNPs/indels are indicated and the percentage sequence identity is calculated alongside the breadth of the BLASTn alignment with respect to the maximum. Total sequenced aligned from the tabulated NUCmer output (NUCmer aligned) alongside the breadth of the NUCmer alignment, is shown. Where relevant, indels that break the alignment are indicated. These are not included in the calculation of sequence identity. b, Pedigree of Lancer (*RHT-B1b*, Australia), Weebill (*RHT-B1b*, Mexico), Claire (*RHT-D1b*, UK) and Mace (*RHT-D1b*, Australia) tracing back to the common accession, Daruma (Japan), which is the donor of *RHT-B1b/RHT-D1b* (ref. ^1^). Lines which are derived from Daruma are indicated in yellow, whereas lines with the wild-type *RHT-B1a* and *RHT-D1a* alleles are indicated in teal. Unknown genotypes are in grey. Important accessions in CIMMYT breeding which are shared in the pedigree of the four sequences cultivars included in the tree are indicated with numbers (see Supplementary Note). Pedigree generated with the Helium software^2^. c, Shared haplotype block on chromosome 4B in six of the 15 sequenced cultivars which carry *RHT-B1b* (Jagger, Lancer, Landmark, Robigus, SY-Mattis and Weebill). Diagram shows the relative size of the shared haplotype among cultivars, with *RHT-B1b* indicated by the vertical black line. Due to the difference in scale, detailed breakpoints are indicated diagrammatically, whereas the middle panel shows at actual scale. Haplotype blocks identified in this study are shown in the bottom panel as visualised in [www.crop-haplotypes.com](http://www.crop-haplotypes.com).


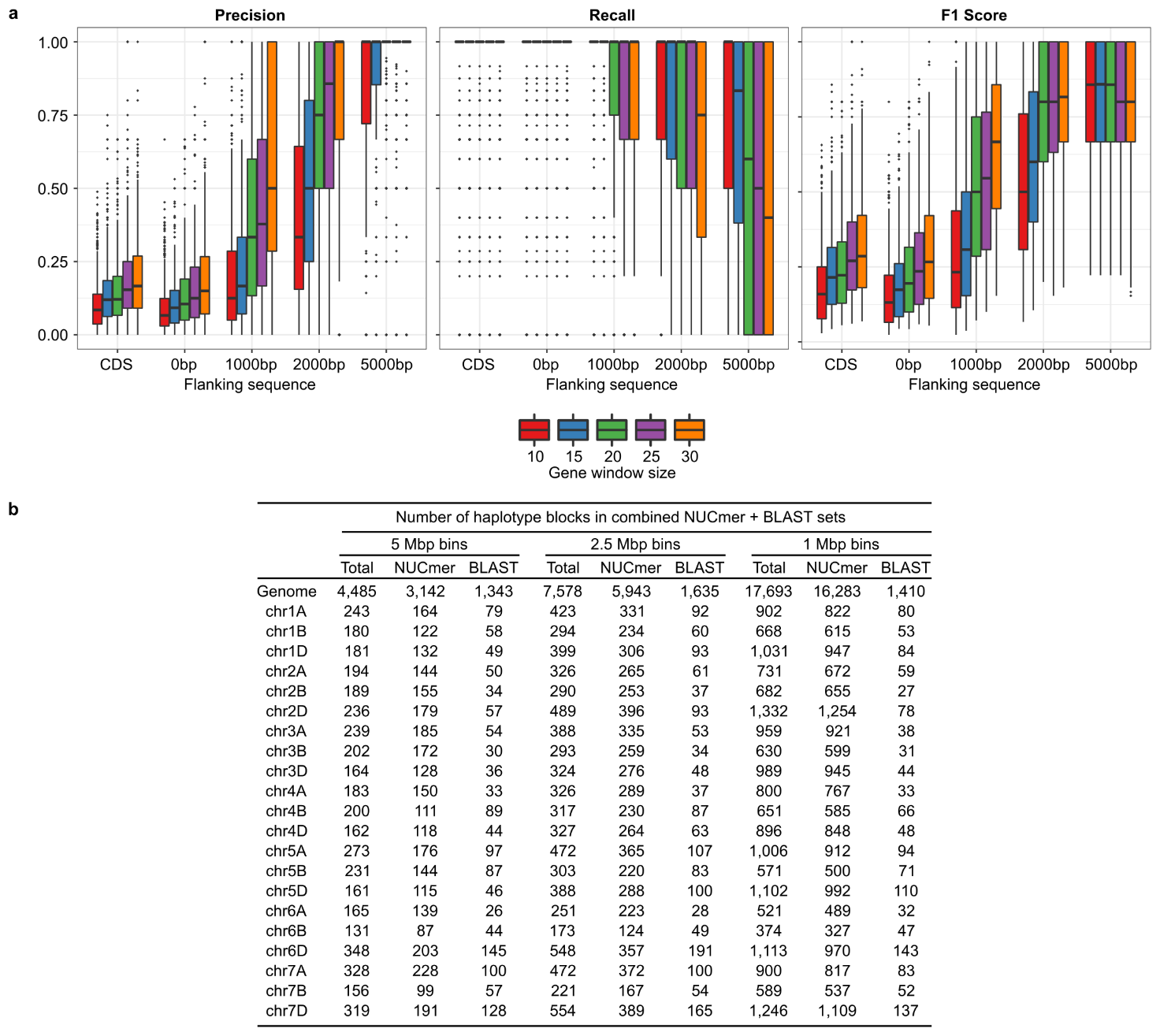


## Supplementary Fig. 3 | Evaluation and integration of NUCmer and BLAST-defined haplotype blocks. a, Precision, recall and F1 score using different flanking sizes (sequence surrounding genes) and window size (number of genes within the sliding window). The NUCmer-defined haplotypes were used as ground truth and compared to the BLAST-defined haplotypes (using different values of the two parameters). The precision, recall, and F1 values were calculated separately for chromosomes 6A, 7A, 2B, 3B, 1D and 4D and then combined. Boxes show interquartile range of data, horizontal line within box indicates median and whiskers are the range of the data with outliers (beyond 1.5-times interquartile range) shown as individual points. b, Number of haplotype blocks identified by NUCmer, BLAST and combined data for each chromosome using 5-, 2.5- and 1-Mbp bins. Note that BLAST blocks listed are those added to the combined dataset and are not all the BLAST blocks called at the initial stage (see Methods).


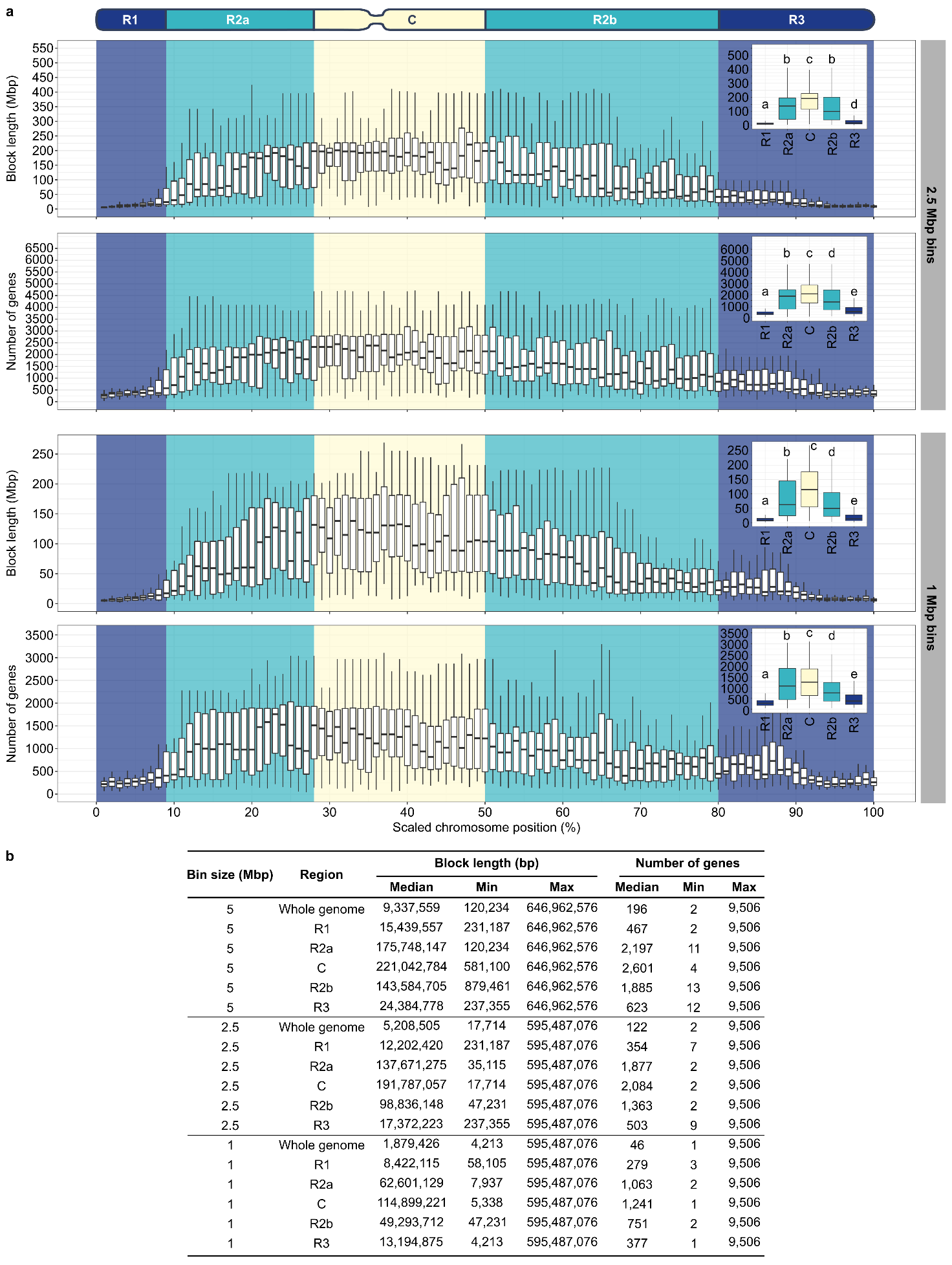


## Supplementary Fig. 4 | Characteristics of haplotype blocks across different bin sizes. a, Length and gene number of haplotype blocks based on 2.5- (middle) and 1-Mbp (bottom) bins sampled at 500 Kbp intervals across all 21 chromosomes (positions scaled to % of maximum chromosome length). Boxplots show distributions of 1% bins. Insets show boxplots for chromosomal compartments (illustrated at top) as previously defined^3^ based on recombination rates (R1+R3 > R2a+R2b > C). Pairwise Wilcox test with Benjamini–Hochberg multiple testing correction was used for statistical analysis. b, Statistics on block size and gene number for the 5-, 2.5- and 1-Mbp bin sizes across different chromosome regions.


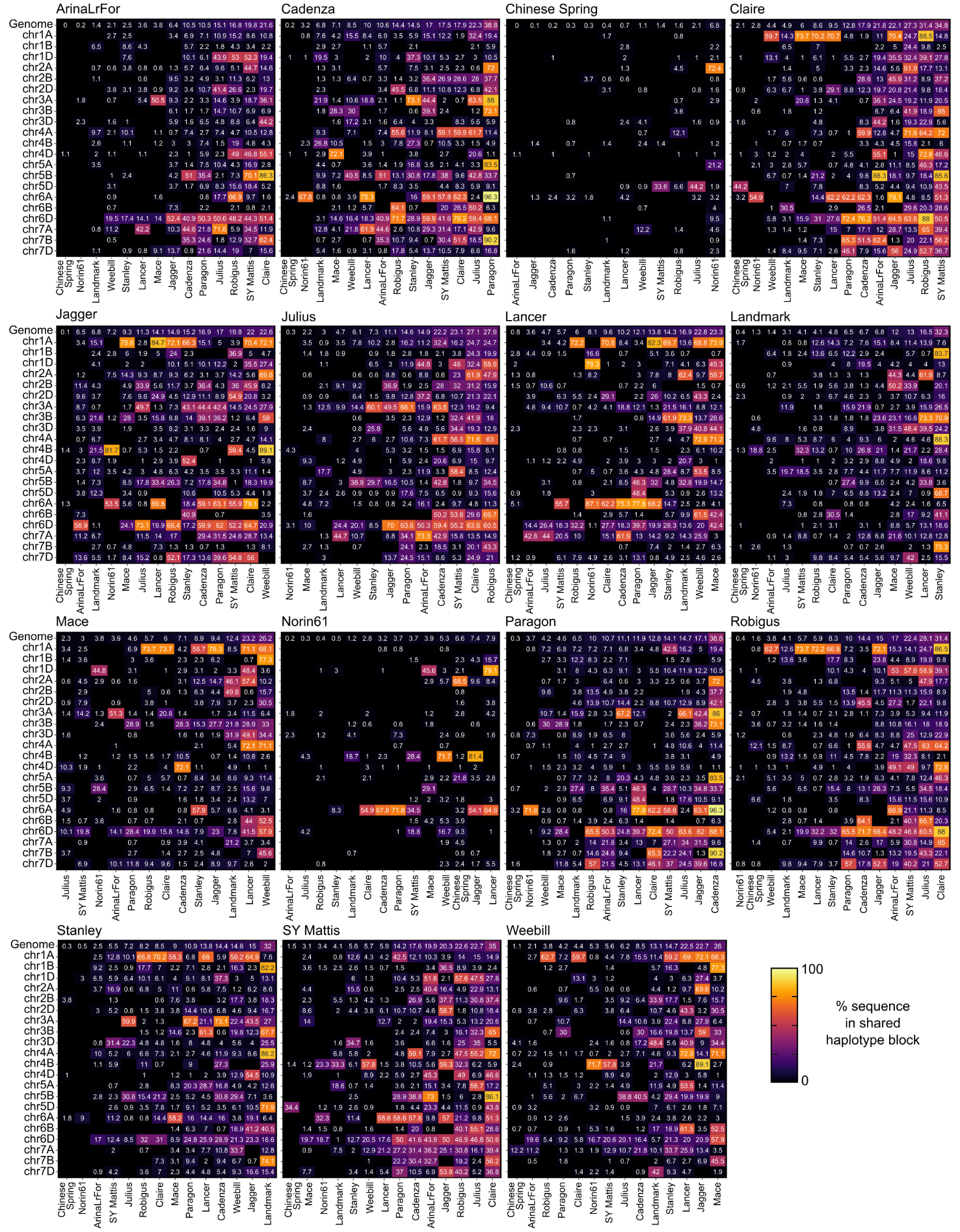


## Supplementary Fig. 5 | Shared common haplotypes among cultivars. Heatmaps for percentage of shared haplotypes in pairwise comparisons between all cultivars for each chromosome (based on 5-Mbp bins). Reference cultivar is indicated on top left of each panel and cultivars being compared to are indicated at the bottom. Cultivars originate from Australia (Mace, Lancer), Canada (Stanley, Landmark), Germany (Julius), Japan (Norin61), Switzerland (ArinaLrFor), USA (Jagger), France (SY Mattis), UK (Cadenza, Paragon, Robigus, Claire) and Mexico (Weebill). Pedigree information for all lines in the Helium format is provided in Supplementary Data 3.


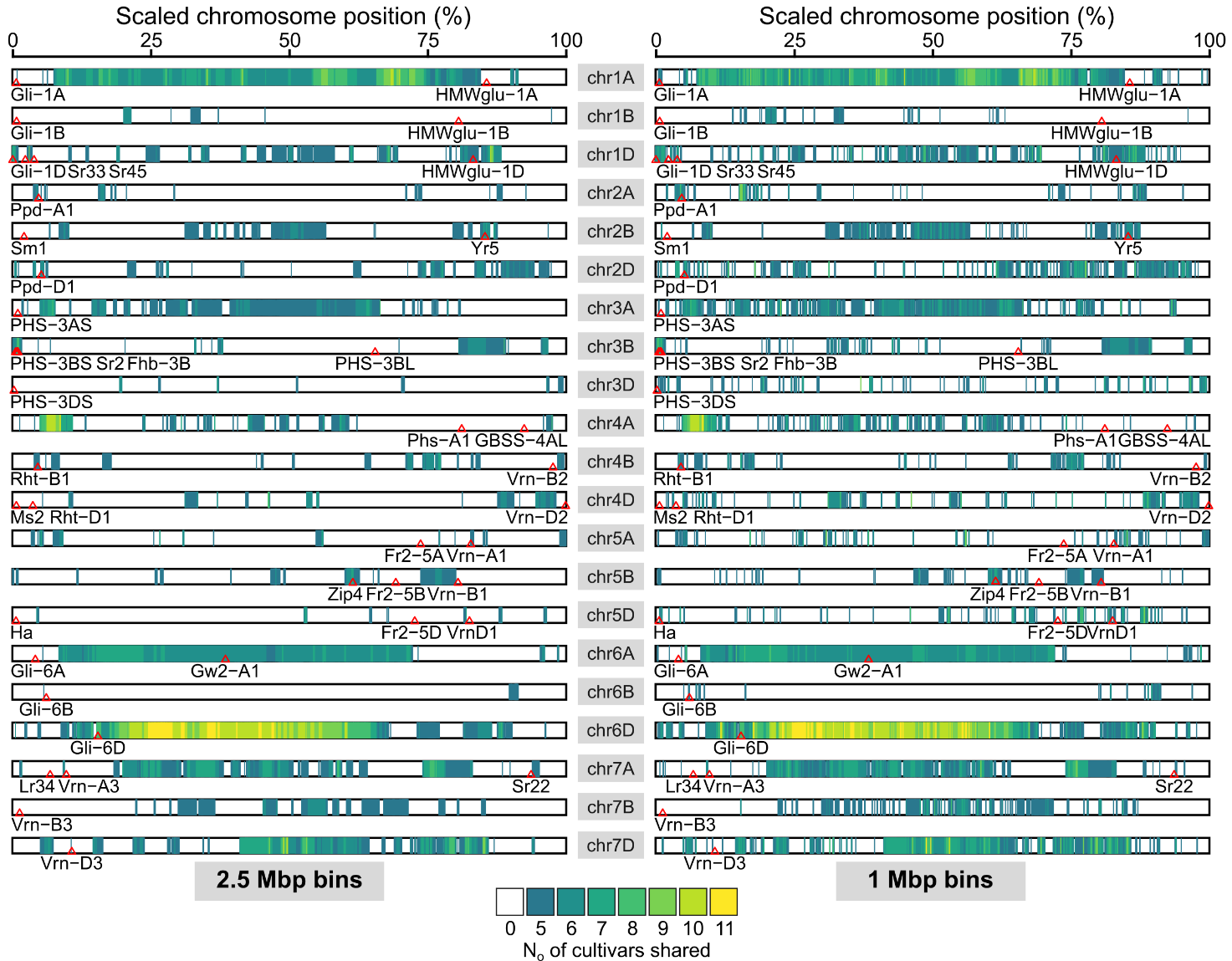


## Supplementary Fig. 6 | Highly conserved haplotypes. Summary of ‘highly conserved’ haplotype blocks across all cultivars (shared with ≥ 5 cultivars; positions scaled to % of maximum chromosome length per cultivar). Values based on haplotype blocks called using 2.5-Mbp (left) and 1-Mbp (right) bins (Supplementary Data 4). Genes of agronomic importance in wheat are indicated by red arrowheads (see Methods).


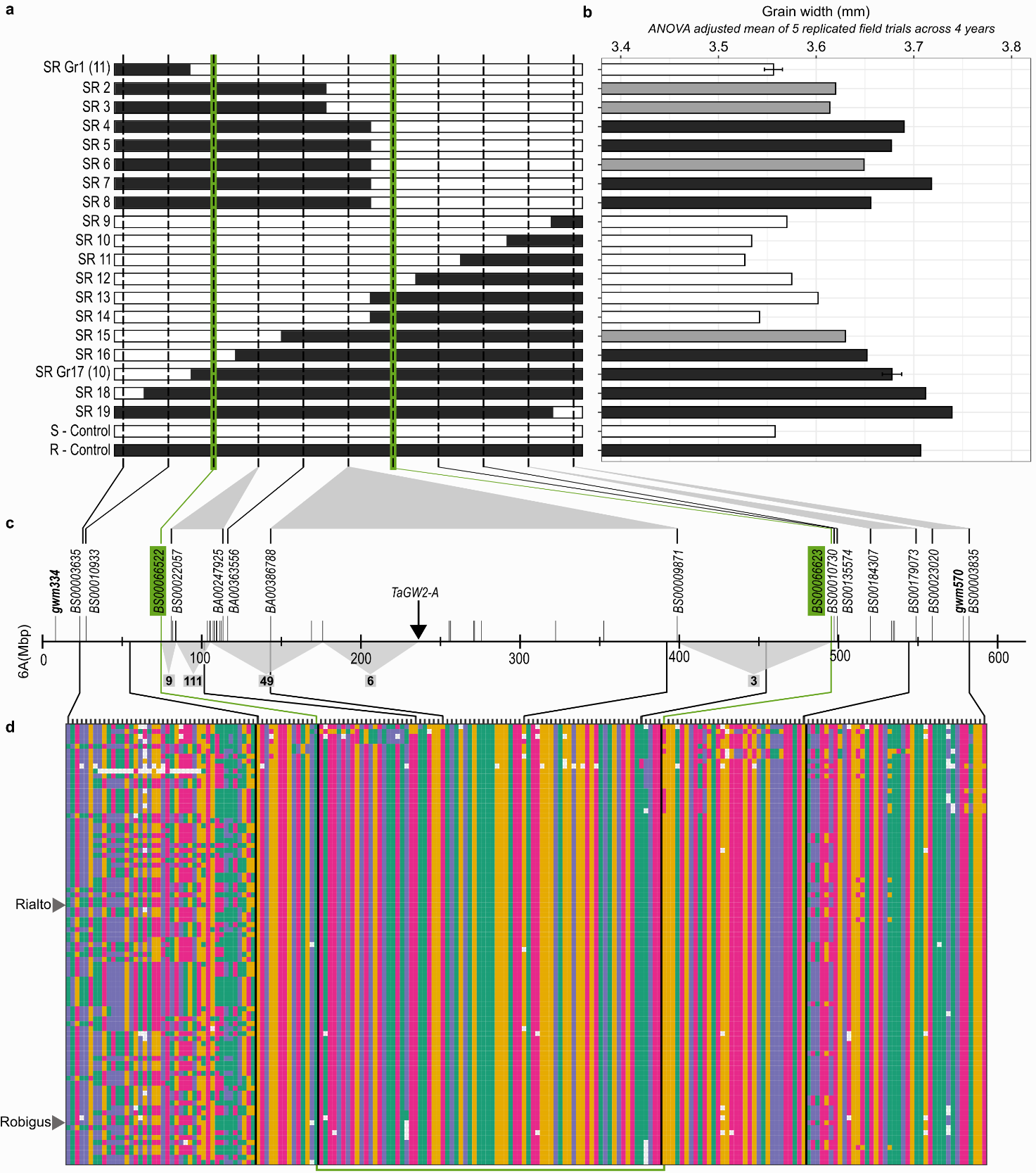


## Supplementary Fig. 7 | Breeders maintain intact chromosome 6A haplotype to maximise phenotypic expression. a, Graphical genotypes of Spark (Haplotype 2, white) x Rialto (Haplotype 3, black) homozygous BC_4_ recombinant inbred lines (RIL). Vertical dashed lines correspond to genetic markers across the interval with the physical position shown in c. For each RIL, white bars represent Spark markers and black bars represent Rialto markers. Markers highlighted in green are the flanking markers for the grain width interval. RILs with recombination outside this region were grouped (if n ≥ 2 independent RILs; GR1 and GR10) with the number of independent RILs in each group indicated by the number in brackets. Parental near-isogenic lines^4^ are shown as S-Control and R-Control, respectively for Spark and Rialto. b, ANOVA adjusted mean for grain width (mm) for each RIL across five trials in four years of replicated field trials (see Methods). For GR1 and GR10 error bars are the standard error of all lines within the RIL group. Bars are coloured according to RIL classification based on ANOVA followed by the Dunnett’s test (*p* < 0.05, see Methods). White indicates significantly similar to the S-control (Spark, H2) and significantly different to the R-control (Rialto, H3), whereas black indicates significantly similar to the R-control (Rialto, H3) and significantly different to the S-control (Spark, H2). Grey correspond to RILs classified as intermediate (see Methods). c, Physical map of chromosome 6A. Markers used in a are positioned based on their physical position on RefSeqv1, with flanking markers highlighted in green. The two additional markers in bold text (*gwm334* and *gwm570*) correspond to the genetic interval for grain width defined previously^4^. Markers which were completely linked in the first recombinant screen to generate the RILs used for phenotyping (a, b) are indicated by a light grey triangle which connects the single genetic position to the wider physical interval. Additional linked markers within these regions are shown as vertical lines in the physical map but are not labelled. Grey triangles pointing downwards indicate the number of additional RILs identified in the second recombinant screen (see Methods) between the genetic markers at the vertices of the triangle. The physical position of *TaGW2-A* (237 Mbp) is indicated by the arrow. d, Allele calls for markers spanning chromosome 6A for 88 UK Recommended List cultivars carrying H3 based on 35K Breeders’ SNP array; A (purple), C (pink), G (green) and T (yellow). Physical positions of landmark markers are indicated by connections to c. Of the 88 lines which carry H3 (Robigus and Rialto labelled as representatives) based on the 35K data (and confirmed by haplotype-informed markers, Supplementary Data 8), 77 have the complete H3 haplotype from markers *BS00066522* to *BS00066623* (74.5 to 496.3 Mbp, 4,731 genes (1587 HC + 3144 LC); highlighted in green). Extending further to markers at 55.7 and 539.6 Mbp (483.9 Mbp, 6,018 genes (2136 HC + 3882 LC)), 70 of the 88 cultivars carry the intact haplotype H3 block.


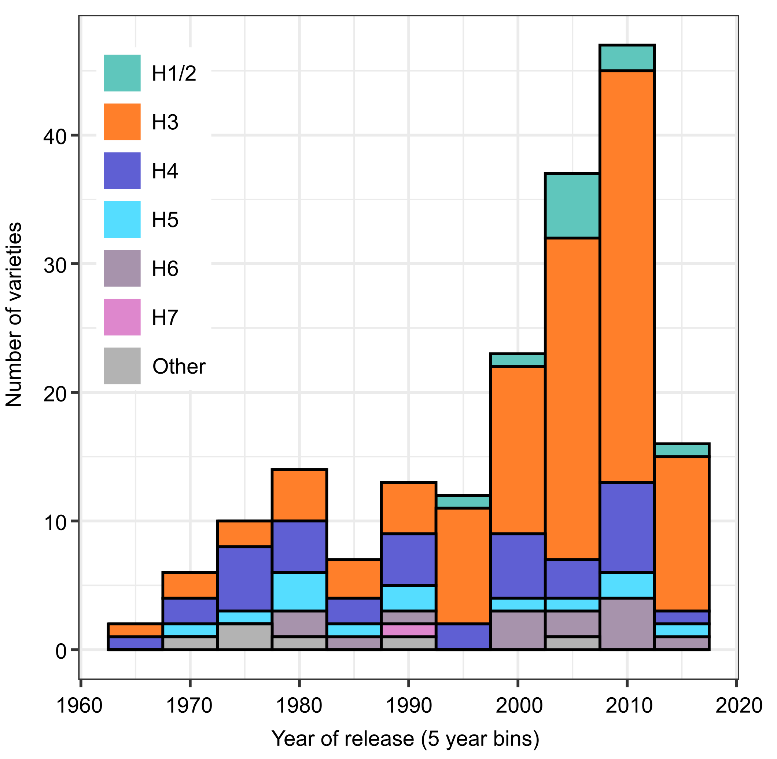


## Supplementary Fig. 8 | 6A haplotypes in breeding germplasm across time. Frequency of chromosome 6A haplotypes in European winter wheat elite cultivars based on year of release. Cultivars were classified based on 15k iSelect data^5^ and binned into 5-year intervals. Lines released 2000 onwards have a higher frequency of haplotype H3 than those lines released before 2000 (χ^2^ = 13.6; df=1, *p* <0.001).


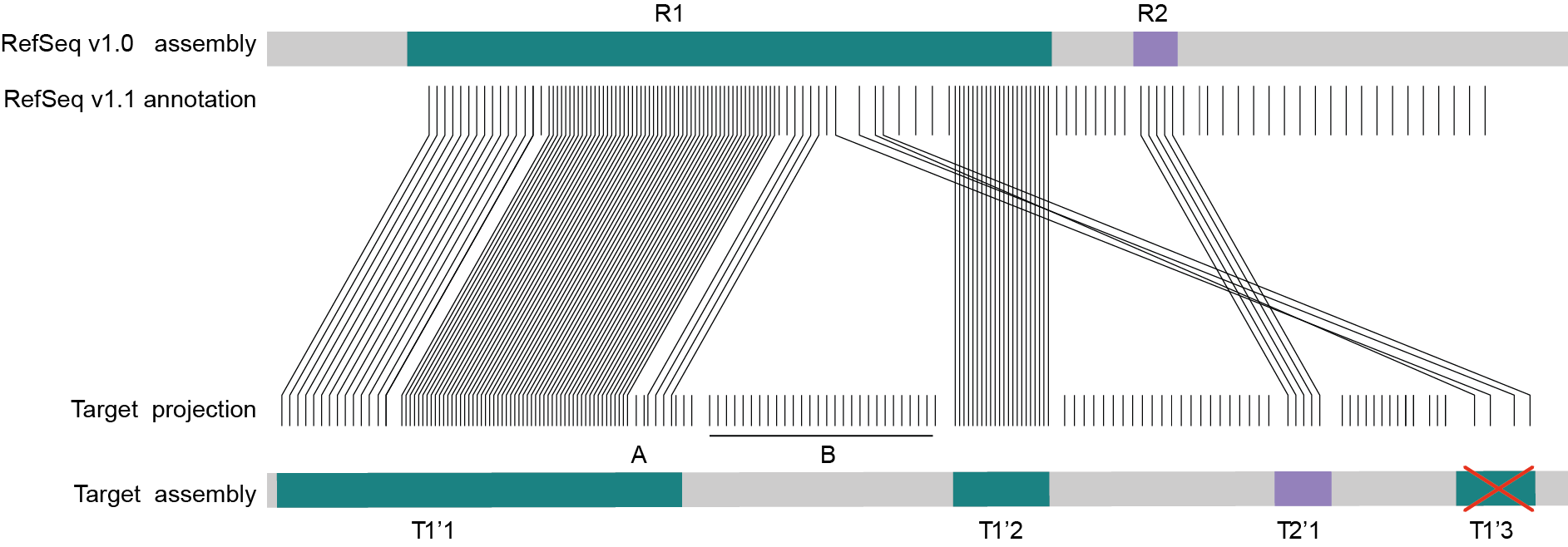


## Supplementary Fig. 9 | Block coordinate conversion between assemblies. The genes in region R1 are projected in Regions T1’1, T1’2 and T1’3. T1’1 has a gene (A) not from the haplotype block, but a single extra gene doesn’t break the projection. There is a stretch of over 20 genes (B) between T1’1 and T1’2 which breaks the block R1. Likewise, region T1’3 is shorter than the minimum 10 genes to consider a block and there are other projected blocks from R1, hence T1'3 is removed. R2 is projected to T2’1, which contains less than the minimum 20 genes required to keep a projection. However, T2’1 is kept as it is the only possible projection for R2.

## Supplementary Table 1 | Watkins x Paragon bi-parental populations. Populations with QTL that span the 6A minimum haplotype block and in which the Watkins allele gives the increasing effect. Traits included are height (cm), grain dimensions (mm or mm^2^), grain weight (g) and yield (kg per plot). Additive effect is reported, alongside absolute phenotypic effect and percentage with respect to population mean.

# Supplementary Note

## Pedigree and sequence analysis of *RHT-B1* and *RHT-D1*

The Green Revolution GA-insensitive *RHT-B1b* and *RHT-D1b* alleles both originate from a single wheat cultivar, Daruma^1^. This line was a parent of Norin10 (Tohoku_34), which was used extensively by CIMMYT to develop semi-dwarf wheat cultivars based on the selection of either the *RHT-B1b* or *RHT-D1b* allele. Lines that carried both semi-dwarfing alleles were too short and were usually not released as cultivars. Norin10 was the *RHT-B1b*/*RHT-D1b* donor to cultivars that were then extensively used by CIMMYT and breeders worldwide to introduce the semi-dwarfing trait into their local cultivars. These cultivars include Penjamo-T-62 (*RHT-B1b*), Lerma Rojo 64 (*RHT-B1b*), Sonora 64 (*RHT-D1b*) and Ciano 67 (*RHT-D1b*). These four cultivars are important parents of several of the cultivars studied here, irrespective of their country of origin, growth habit (winter or spring sown) and *RHT* allelic status. For example, Claire a UK winter wheat cultivar that carries *RHT-D1b* has Penjamo-T-62, Sonora64 and Ciano67 in its pedigree, similar to three spring cultivars that all carry the *RHT-B1b* allele; Lancer (Australia), Landmark (Canada) and Weebill (Mexico). This highlights the complex inter-crossing and selection history of most modern wheat cultivars. The pedigrees of Weebill, Lancer, Claire and Mace are shown in Supplementary Fig. 2b.

The pedigrees also point to the identical-by-descent nature of the *RHT-B1b* and *RHT-D1b* haplotypes in the cultivars that carry these alleles, as they all can be traced back to the common wheat line Daruma. This allowed us to investigate the wider physical sequence and determine the extent of conservation among lines that share the same *RHT-B1b* or *RHT-D1b* alleles.

*RHT-B1b*: Six of the 15 sequenced cultivars carry the *RHT-B1b* allele (Jagger, Lancer, Landmark, Robigus, SY_Mattis and Weebill). This ‘highly conserved’ haplotype surrounding *RHT-B1b* is denoted by a red arrowhead in Fig. 2c. Using the haplotype blocks, we identified the extent to which the *RHT-B1b* haplotype is shared to determine the length of each of these regions in the six cultivars. Robigus (5.8 Mbp) and Lancer (18.5 Mbp) carry the smallest common haplotypes of the group, followed by Landmark (151 Mbp) and SY-Mattis (395 Mbp). The shared haplotype block between Jagger and Weebill extends further and encompasses 595 to 601 Mbp in these cultivars (Supplementary Fig. 2c). In other words, the impact of the introduction of *RHT-B1b* in Jagger and Weebill means that these two cultivars share 88.5% and 89.3% of their chromosome 4B physical sequence, whereas Robigus and Lancer share just 0.9% and 2.8%, respectively (including *RHT-B1b*). The progressive decrease in shared sequence between these cultivars is well visualised in Fig. 2b where the most highly shared sequence across chromosome 4B is at *RHT-B1b* (32.056 Mbp in Jagger) and then progressively decreases as the comparison extends across the chromosome.

*RHT-D1b*: Four of the 15 sequenced cultivars carry the *RHT-D1b* allele (Claire, Julius, Mace and Norin61). In contrast to *RHT-B1b*, the extent of the shared *RHT-D1b* haplotype block is much smaller in these cultivars. The largest shared block is between Mace and Claire (~15 Mbp) whereas Julius only shares 3.7 Mbp with the other cultivars (including *RHT-D1b*).

# References

1 Wilhelm, E. P., Boulton, M. I., Barber, T. E. S., Greenland, A. J. & Powell, W. Genotype analysis of the wheat semidwarf *Rht-B1b* and *Rht-D1b* ancestral lineage. *Plant Breeding* **132**, 539-545 (2013).

2 Shaw, P. D., Graham, M., Kennedy, J., Milne, I. & Marshall, D. F. Helium: visualization of large scale plant pedigrees. *BMC Bioinformatics* **15**, 259 (2014).

3 Appels, R. *et al.* Shifting the limits in wheat research and breeding using a fully annotated reference genome. *Science* **361**, eaar7191 (2018).

4 Simmonds, J. *et al.* Identification and independent validation of a stable yield and thousand grain weight QTL on chromosome 6A of hexaploid wheat (*Triticum aestivum* L.). *BMC Plant Biol.* **14**, 191 (2014).

5 Voss-Fels, K. P. *et al.* Breeding improves wheat productivity under contrasting agrochemical input levels. *Nat. Plants* **5**, 706-714 (2019).
