# Supplementary material for: A haplotype-led approach to increase the precision of wheat breeding
Source: Commun Biol. 2020 Nov 25;3:712. doi: 10.1038/s42003-020-01413-2 (PMC7689427; doi:10.1038/s42003-020-01413-2)
Supplement: Supplementary file 2 — Description of Additional Supplementary Files [file 42003_2020_1413_MOESM2_ESM.pdf]

Description of Additional Supplementary Files

File Name: Supplementary Data 1
Description: **10 Wheat Genome Project consortium members**

File Name: Supplementary Data 2
Description: **Final haplotype blocks**
Combined sets of haplotype blocks called using NUCmer and BLAST approaches using 5, 2.5 and 1 Mbp bins (blocks called at different levels of binning are shown on separate sheets).

File Name: Supplementary Data 3
Description: **Helium pedigree file**
Pedigree information for all sequenced cultivars included in the haplotype analysis.

File Name: Supplementary Data 4
Description: **Highly conserved chromosome regions**
Regions identified as 'highly conserved' based on the sharing of a common haplotype with five or more other cultivars.

File Name: Supplementary Data 5
Description: **Previously identified QTL on chromosome 6A**
IWGSC RefSeqv1.0 coordinates of 6A productivity-related QTL presented in Fig. 3a.

File Name: Supplementary Data 6
Description: **Markers used to characterise 6A recombinants**
Names, primer sequences and IWGSC RefSeqv1.0 positions of KASP markers used to characterise 6A recombinants.

File Name: Supplementary Data 7
Description: **Grain width phenotypes of 6A recombinants**
ANOVA adjusted means, Dunnett's test allocations and details of recombinants grown in each field trial (sheet 1) and raw grain width phenotype data (sheet 2) for 6A recombinants.

File Name: Supplementary Data 8
Description: **Haplotype allocation based on published markers**
Genotyping data for germplasm presented in Fig. 4 across the 6A minimum haplotype block (187–445 Mbp) from the 15K iSelect, 35 breeders' Axiom and exome-capture data (post-filtering, see Methods), shown on separate sheets. All markers on chromosome 6A are provided for the 35K breeders' Axiom array.

File Name: Supplementary Data 9
Description: **Haplotype-informed primers and genotypes**
Names, KASP primer sequences and IWGSC RefSeqv1.0 positions of haplotype-informed SNP markers across the 6A minimum haplotype block and genotypes for all 15 sequenced cultivars.

File Name: Supplementary Data 10

Description: **Watkins and UK RL haplotype assignment using haplotype-informed** Markers Genotype calls and haplotype allocation across the 6A minimum haplotype block based on haplotypeinformed markers for Watkins landraces and UK Recommended List cultivars
